# Tapping Culture Collections for Fungal Endophytes: First Genome Assemblies for Three Genera and Five Species in the *Ascomycota*

**DOI:** 10.1093/gbe/evad038

**Published:** 2023-03-07

**Authors:** Rowena Hill, Quentin Levicky, Frances Pitsillides, Amy Junnonen, Elena Arrigoni, J Miguel Bonnin, Anthony Kermode, Sahr Mian, Ilia J Leitch, Alan G Buddie, Richard J A Buggs, Ester Gaya

**Affiliations:** Royal Botanic Gardens Kew, Richmond, UK; School of Biological and Behavioural Sciences, Queen Mary University of London, London, UK; Department of Molecular Biology and Biotechnology, The University of Sheffield, Sheffield, UK; Royal Botanic Gardens Kew, Richmond, UK; Royal Botanic Gardens Kew, Richmond, UK; Royal Botanic Gardens Kew, Richmond, UK; CABI, Bakeham Lane, Egham, UK; CABI, Bakeham Lane, Egham, UK; Royal Botanic Gardens Kew, Richmond, UK; Royal Botanic Gardens Kew, Richmond, UK; CABI, Bakeham Lane, Egham, UK; Royal Botanic Gardens Kew, Richmond, UK; School of Biological and Behavioural Sciences, Queen Mary University of London, London, UK; Royal Botanic Gardens Kew, Richmond, UK

**Keywords:** *Ascomycota*, culture collections, cytometric completeness, fungal endophytes

## Abstract

The *Ascomycota* form the largest phylum in the fungal kingdom and show a wide diversity of lifestyles, some involving associations with plants. Genomic data are available for many ascomycetes that are pathogenic to plants, but endophytes, which are asymptomatic inhabitants of plants, are relatively understudied. Here, using short- and long-read technologies, we have sequenced and assembled genomes for 15 endophytic ascomycete strains from CABI’s culture collections. We used phylogenetic analysis to refine the classification of taxa, which revealed that 7 of our 15 genome assemblies are the first for the genus and/or species. We also demonstrated that cytometric genome size estimates can act as a valuable metric for assessing assembly “completeness”, which can easily be overestimated when using BUSCOs alone and has broader implications for genome assembly initiatives. In producing these new genome resources, we emphasise the value of mining existing culture collections to produce data that can help to address major research questions relating to plant–fungal interactions.

**Significance**

Historically, efforts aimed at whole-genome sequencing of fungi have been biased towards economically important plant pathogens, but improving genomic resources of commensal and mutualistic fungi is fundamental if we are to fully understand the whole range of plant–fungal interactions. In generating these new genome assemblies for fungal endophytes—asymptomatic inhabitants of plants—we provide valuable new resources for exploring the pathogenic–mutualistic spectrum in different lineages across the *Ascomycota*. Our results demonstrate the value of mining existing culture collections to produce much-needed genomic data for neglected lineages of plant-associated fungi.

## Introduction

To date, most fungal genome sequencing effort has been skewed towards pathogens and, of those, plant pathogens (Aylward 2017), but recent and ongoing initiatives are rapidly increasing the number of genome assemblies available for nonpathogenic strains, such as commensal or mutualistic plant-associated fungi (https://jgi.doe.gov/our-projects/csp-plans/). Improving genomic resources for nonpathogenic relatives of phytopathogens is key to understanding functional differences between different forms of plant associated lifestyles, and will allow us to explore how and why plant–fungal interactions evolve. This is particularly crucial for fungal endophytes, asymptomatic plant inhabitants which predominantly belong to the phylum *Ascomycota* (Rodriguez et al. 2009; Hardoim 2015). Factors controlling whether a fungus exhibits endophytism versus pathogenicity are not well defined. Case-study comparisons between closely related pathogens and endophytes have started to reveal lineage-specific patterns or mechanisms that may contribute to lifestyle (Hacquard 2016; Niehaus 2016; Stauber et al. 2020; Hill et al. 2022), however we have no indication of whether they will hold true for all ascomycete endophytes, which are spread across the entire phylum (Huang 2018; U’Ren 2019). If we are to better understand endophytism, and therefore improve the chance of predicting the pathogenic potential of fungal strains, comparisons across a broader taxonomic scale are needed. This is only achievable through the generation of new, high-quality genome assemblies for endophyte strains.

Culture collections are a powerful resource for addressing all manner of research questions. The CABI collection (Egham, UK) is one of the world’s largest fungal culture collections, boasting 28,000 strains spanning 100 years and 142 countries (Smith et al. 2022). Here, we capitalised on endophytic strains deposited in CABI’s collection to successfully sequence, assemble and annotate genomes for 15 taxa across 8 families and 5 orders. Where possible, we additionally produced cytometric genome size estimates for stringent quality assessment of these new genome assemblies (Hill et al. 2021a).

For new genomic resources to be of use to the science community, it is of major importance to ensure accurate identification and classification of taxa. Phylogenetics has become an essential step in fungal classification, not least when dealing with cultured microfungi where morphological features are often particularly challenging to study and can be less informative, or not informative at all, for distinguishing species or even genera (Crous and Groenewald 2005; Shivas and Cai 2012). Phylogenetic analyses revealed our assemblies to be the first for three ascomycete genera—*Collariella*, *Neodidymelliopsis* and *Neocucurbitaria*—and five species—*Ascochyta clinopodiicola*, *Didymella pomorum*, *Didymosphaeria variabile*, *Neocosmospora piperis* and *Neocucurbitaria cava*. Four more taxa—*Didymella* sp. IMI 355093, *Gnomoniopsis* sp. IMI 355080, cf. *Kalmusia* sp. IMI 367209 and *Neurospora* sp. IMI 360204—require additional assessment to determine whether they are new or previously described species, but based on existing data they also likely represent the first genome assemblies for their to-be-assigned species. As well as providing the first genomic resources for taxa, these endophyte assemblies will enable future work comparing endophytic and phytopathogenic strains widely across the *Ascomycota*.

## Results and Discussion

### Genome Assemblies

**Fig. 1.**
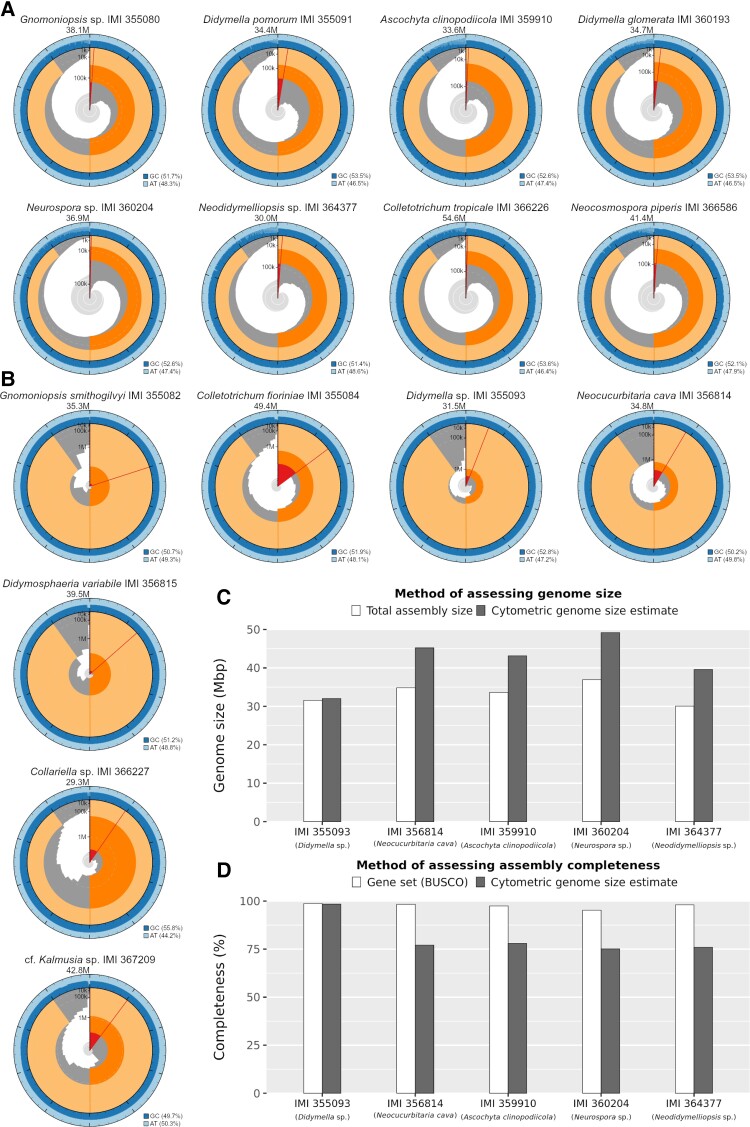
Snail plots produced using BlobToolKit v3.4.0 (Challis et al. 2020) summarising assembly contiguity for (*A*) short-read and (*B*) hybrid assemblies. The distribution of fragment lengths is shown in dark grey with the plot radius scaled to the longest fragment of the assembly, shown in red. The pale grey spiral shows the cumulative fragment count on a log scale. The orange and cream arcs show the N50 and N90 fragment lengths. The outside blue bands show the distribution of GC/AT content. (*C*) Total genome size as indicated by total assembly length versus cytometric genome size estimation. (*D*) Genome assembly completeness as measured by gene set (BUSCOs) versus cytometric genome size estimation.

We report 15 endophyte assemblies here—8 using Illumina short-reads and 7 with additional Oxford Nanopore Technologies long-reads for hybrid assembly. We tested three assembly tools for both approaches to ensure we produced the highest quality assembly. For the short-read assemblies, SPAdes (Bankevich 2012) consistently produced assemblies with the best contiguity and completeness statistics compared with ABySS (Simpson 2009) and MEGAHIT (Li 2016), however, for hybrid assemblies, hybridSPAdes resulted in markedly worse contiguity than either Flye (Kolmogorov et al. 2019) or Raven (Vaser and Šikić 2021) (supplementary fig. 1, Supplementary table 1). There was little difference in the performance of Flye and Raven, although Raven produced the “best” assembly for five out of seven strains (table 1). Unsurprisingly, incorporating long-reads resulted in much less fragmented assemblies, some likely approaching chromosome-level (fig. 1*A*,*B*).

**Table 1:** Statistics for the 15 new endophyte assemblies. See supplementary table 1 for comparisons of statistics across all assembly tools. SR, short-read; LR, long-read

							QUAST				BUSCO	Funannotate
	IMI	Name	Tool	Coverage (SR)	Coverage (LR)	Contam. (%)	# contigs ≥500bp	Length (Mbp)	GC (%)	N50	Completeness (%)	# genes
Short-read	355080	*Gnomoniopsis* sp.	SPAdes	112×	—	7.3	694	38.08	51.69	127,272	93.14	10,907
	355091	*Didymella pomorum*	SPAdes	252×	—	2.9	524	34.42	53.52	218,427	98.94	11,427
	359910	*Ascochyta clinopodiicola*	SPAdes	139×	—	2.6	1,199	33.61	52.55	73,892	97.48	10,203
	360193	*Didymella glomerata*	SPAdes	253×	—	2.3	724	34.73	53.46	179,824	98.42	10,766
	360204	*Neurospora* sp.	SPAdes	122×	—	18.5	3,250	36.93	52.64	25,999	95.25	10,020
	364377	*Neodidymelliopsis* sp.	SPAdes	186×	—	1.5	1,103	30.05	51.51	74,885	98.01	9,755
	366226	*Colletotrichum tropicale*	SPAdes	86×	—	1.2	1,685	54.63	53.59	63,560	96.42	13,995
	366586	*Neocosmospora piperis*	SPAdes	116×	—	2.2	1,248	41.42	52.32	91,570	96.31	12,790
Hybrid	355082	*Gnomoniopsis smithogilvyi*	Flye	113×	44×	12.2	9	35.29	50.70	6,429,383	86.64	10,375
	355084	*Colletotrichum fioriniae*	Flye	193×	20×	0.1	45	49.45	51.93	2,983,733	98.07	12,178
	355093	*Didymella* sp.	Raven	300×	138×	0.0	27	31.53	52.85	1,301,886	98.65	9,918
	356814	*Neocucurbitaria cava*	Raven	160×	165×	0.0	24	34.85	50.24	1,616,366	98.30	11,048
	356815	*Didymosphaeria variabile*	Raven	212×	216×	0.0	11	39.45	51.25	4,705,368	98.12	12,728
	366227	*Collariella* sp.	Raven	316×	39×	0.9	49	29.31	55.80	1,760,284	87.92	8,224
	367209	cf. *Kalmusia* sp.	Raven	208×	27×	0.1	30	42.78	49.69	2,200,773	98.18	13,561

Despite originating from axenic cultures, we still detected some contaminant contigs that were removed from the assemblies. The majority of contaminants belonged to other ascomycete fungi, although there was also some bacterial contamination found (supplementary fig. 2). These contigs generally represented a small proportion of the assemblies, however, in two cases a considerable proportion of the assembly was filtered out: 19% for IMI 360204 and 12% for IMI 355082 (table 1).

### Flow Cytometry Revealed Some Assemblies to be Less Complete than BUSCOs Would Suggest

Genome size measurements were successfully obtained for five of the strains using flow cytometry (supplementary table 2). For these strains, we were able to compare total assembly length against cytometric genome size estimates, which revealed that most assemblies were notably smaller than the “true” genome size (fig. 1*C*) despite having a high percentage of single-copy BUSCOs (fig. 1*D*). The exception was strain IMI 355093 (*Didymella* sp.), which was estimated to be highly complete according to cytometric measurements as well as BUSCOs (fig. 1*D*). These cytometric estimates will provide a benchmark that future attempts to refine these assemblies can be measured against.

### Phylogenetic Analyses Classified Strains as Belonging to 11 Genera, with 9 Strains Resolved to Species-level

We produced multilocus phylogenetic trees using RAxML-NG (Kozlov et al. 2019) to refine the original classifications from CABI’s records. All but one of the taxa were confidently assigned to genus-level and nine to species-level (supplementary fig. 3). The placement of IMI 367209 within the *Didymosphaeriaceae* was ambiguous, as it fell within a poorly supported clade alongside *Kalmusia erioi* and *Kalmusia cordylines*, but the genus *Kalmusia* was not resolved monophyletically (supplementary fig. 3*D*), and so the strain has been conservatively dubbed here as “cf. *Kalmusia* sp.”. Our taxonomic assignments will benefit from validation through updated morphological assessments of the cultures, however the value of these genome assemblies has already been increased considerably with the revised names presented here.

## Materials and Methods

### Extraction and Sequencing of Genomic DNA

The 15 endophyte strains used in this study were obtained from the CABI culture collection (supplementary table 3). All steps involving handling of fungal material were done under sterile conditions. Strains were taken out of cryopreservation and incubated on 2% malt extract agar at 25 °C for 1–2 weeks. A fragment of mycelium was transferred to flasks of 200 ml glucose yeast medium (GYM). Flasks were placed on an orbital shaker for 1 week at 25 °C and shaken at 150 rpm. Mycelium was recovered via vacuum filtration, transferred to an empty petri dish and freeze dried overnight. The lyophilised material was crushed using a mortar and pestle for DNA extraction, which was done using the Qiagen DNeasy Plant Mini Kit (Qiagen, Redwood City, CA, United States) following the manufacturer’s instructions. DNA concentration was quantified with a Quantus™ Fluorometer (Promega, Wisconsin, USA) and purity (260/280 absorbance ratio of approximately 1.8) was assessed with a NanoDrop spectrophotometer (Thermo Fisher Scientific, Massachusetts, USA). To ascertain that DNA had successfully been extracted from the intended strain rather than a contaminant, 0.5 μl of DNA extraction was used for amplification and Sanger sequencing of the ITS barcode, as described by Hill (2021b). ITS sequences were searched against the UNITE database (Nilsson 2019) and the NCBI nucleotide database (https://ncbi.nlm.nih.gov/) via corresponding web blastn services to identify the most similar species hypothesis (SH) for each strain. We additionally corroborated the similarity-based results by placing the ITS sequences in the 6-loci *Pezizomycotina* v2.1 reference tree (Carbone 2017) of T-BAS v2.3 (Carbone 2019) with default settings (supplementary fig. 4).

For short-read sequencing, DNA extractions were sent to Macrogen (Macrogen Inc., South Korea) for library preparation and sequencing: library preparation was performed using the Nextera XT DNA Library Preparation Kit and 151 bp paired-end reads were sequenced using the NovaSeq 6000 platform (Illumina, San Diego, CA, USA). If we were able to extract ≥1 μg of DNA, strains were also processed for long-read sequencing. For each strain, the appropriate volume for 1 μg of DNA was diluted with sterile, nuclease-free water to obtain the required 47 μl of DNA for the library preparation method described here. Half of the DNA solution (23.5 μl) was then sheared to a fragment size of ∼20 Kbp by centrifuging in a g-TUBE (Covaris, Inc., Woburn, MA, USA) at 4,200 rpm for 1 min. Sequencing libraries were prepared from the mixture of sheared and unsheared DNA using the SQK-LSK109 Ligation Sequencing Kit (Oxford Nanopore Technologies Inc., Oxford, UK) following the manufacturer’s Genomic DNA by Ligation protocol (version GDE_9063_v109_revAE_14Aug2019). The Short Fragment Buffer was used during the clean-up step to purify all fragments equally. DNA repair and end-prep was performed using the NEBNext FFPE DNA Repair and Ultra II End Repair/dA-Tailing modules (New England BioLabs, Ipswich, MA, USA). The library was loaded into a FLO-MIN106 flow cell and sequenced with a MinION device (Oxford Nanopore Technologies Inc.) for ∼48 h using the MinKNOW application (Oxford Nanopore Technologies Inc.). Fast basecalling was performed after sequencing using guppy v4.5.3 (Oxford Nanopore Technologies Inc.).

### Cytometric Genome Size Estimation

Where possible, cultures were additionally sampled for flow cytometry 10–56 days after subculturing depending on the growth rate of the sample. *Coprinellus micaceous* (62.60 Mbp/1C) and *Coprinopsis piacea* (52.83 Mbp/1C) were used as internal reference standards. See the Supplementary material for full methodological details; in brief, the preparation of each sample was completed following the One-Step Protocol using LB01 buffer (Doležel et al. 1989), as outlined by Pellicer et al. (2020). We used the Partec FloMax v2.4d software (Sysmex Partec GmbH) to produce histograms showing the relative fluorescence of nuclei (supplementary fig. 5) and the holoploid 1C genome size of each strain was estimated using the following formula:


$$\frac{{\text{Mean G}}_{1}\;\text{fluorescence peak of sample}\times \text{1C nuclear DNA content of reference standard}}{{\text{Mean G}}_{1}\;\text{fluorescence peak of reference standard}}$$

#### De Novo Genome Assembly

For strains which only had short-read data, the assembly pipeline from Hill et al. (2022) was used, comparing ABySS v2.0.2 (Simpson 2009), MEGAHIT v1.2.9 (Li 2016) and SPAdes v3.11.1 (Bankevich 2012). If we were also able to obtain long-read sequence data for strains, hybrid assembly was performed with comparison across three tools: Flye v2.6 (Kolmogorov et al. 2019), Raven v1.6.1 (Vaser and Šikić 2021), and hybridSPAdes v3.11.1 (Antipov et al. 2016). The former two methods involved assembly using only the raw long-reads, before mapping the short-reads onto the resulting contigs using BWA-MEM v0.7.17-r1188 (Li 2013) in order to polish with Pilon v1.2.4 (Walker 2014). In contrast, hybridSPAdes used both long- and short-reads to construct contigs, before similarly polishing with the short-reads using BWA-MEM and Pilon. For Flye, which requires an estimate of total genome size, cytometric genome size estimates described above were used where possible, otherwise the average genome size for the order according to Hill et al. (2021a) was used.

### Quality Assessment and Contaminant Removal

To select the “best” assembly across the different assembly tools, contiguity was assessed using QUAST v5.0.2 (Gurevich et al. 2013) and completeness was assessed with BUSCO v3.0.1 (Simão et al. 2015) using the ascomycota_odb10.2020-09-10 lineage dataset of 1,706 single-copy orthologues. BlobTools v1.1 (Laetsch and Blaxter 2017) was used to check for possible contamination in the best assemblies. To create hit files, contigs were searched against the UniRef90 database downloaded on August 9, 2022 (Suzek 2015) using DIAMOND v2.0.15.153 (Buchfink et al. 2021) and against the NCBI nucleotide database downloaded on August 17, 2022 using BLAST+ v2.11.1 (Camacho 2009). To create BAM files of mapped reads, long-reads were mapped back onto hybrid assemblies using minimap2 v2.5 (Li 2018), while short-reads were mapped back onto short-read assemblies using BWA-MEM v0.7.17-r1188 (Li 2013). Hit and BAM files were then used by BlobTools to create BlobPlots. Contigs that were not assigned to the correct taxonomic class and contigs with a coverage of less than 10× were removed from assemblies using seqtk v1.2-r94 (https://github.com/lh3/seqtk). Mitochondrial and adapter contamination flagged by NCBI during the assembly submission process was trimmed using bedtools v2.28.0 (Quinlan and Hall 2010). QUAST and BUSCO were then run again on the contamination-filtered assemblies to produce final quality statistics.

### Assembly Annotation

A *de novo* repeat library was generated for the selected assembly for each strain with RepeatModeler v2.0.1 (Smit and Hubley 2015) and used as a custom library for softmasking with RepeatMasker v4.0.9 (Smit et al. 2015). Masked assemblies were structurally annotated using the Funannotate v1.8.12 pipeline (Palmer and Stajich 2020). Proteins and EST clusters of closely related taxa were downloaded from MycoCosm (Grigoriev 2014) to inform gene prediction—taxa are listed in the Supplementary material. We used the funannotate predict command to train and run three *ab initio* gene predictors—AUGUSTUS v3.3.2 (Stanke 2006), GlimmerHMM (Majoros et al. 2004) and SNAP v2006-07-28 (Korf 2004)—and output consensus gene models according to EVidenceModeler v1.1.1 (Haas 2008).

Functional prediction of the gene models was performed using InterProScan v5.57-90.0 (Jones 2014) with mapping to gene ontology terms; eggNOG-mapper v2.1.9-4dfcbd5 (Cantalapiedra et al. 2021) based on the eggNOG orthology database v5.0.2 (Huerta-Cepas 2019), with sequence searches using DIAMOND v2.0.15; and antiSMASH v6.1.1 (Blin 2021). The funannotate annotate command was then used to map the results onto the assembly annotations, with additional searches against UniProt v2022_02 (Bateman 2021), MEROPS v12 (Rawlings et al. 2012), dbCAN v10.0 (Yin 2012), and BUSCO dikarya gene models. Misannotations that were flagged by NCBI during the assembly submission process were manually checked and edited.

### Phylogenetic Analysis

Using our results from UNITE, NCBI, and T-BAS (supplementary fig. 4) to guide taxon sampling, we searched the literature for existing phylogenies and available genetic marker sequences for the different lineages to which our samples potentially belonged (Nygren 2011; Wang 2016, 2022; Chen et al. 2017; Wanasinghe 2017; Crous 2019, 2021; Jaklitsch 2018; Valenzuela-Lopez 2018; Hyde 2019; Hou 2020; Scarpari 2020; Vieira 2020; Jiang 2021; Karácsony et al. 2021; Liu 2022; Wanasinghe and Mortimer 2022). Various combinations of 13 genetic markers were selected for the different lineages, sequences for which were retrieved from GenBank (supplementary table 4). A new script, GenePull (https://github.com/Rowena-h/MiscGenomicsTools/tree/main/GenePull), was created to extract sequences for each of the selected markers from our own genome assemblies using blastn and bedtools (Quinlan and Hall 2010).

We aligned each gene separately for the different lineages using MAFFT v7.480 (Katoh and Standley 2013) and manually checked the gene alignments before trimming using trimAl v1.4.rev15 (Capella-Gutiérrez et al. 2009) with the -gappyout option. As multiple LSU copies were extracted from the *Didymosphaeriaceae* assemblies, all of the copies were included in the *Didymoshaeriaceae* LSU alignment. A gene tree was estimated for the LSU alignment using RAxML-NG v1.0.1 (Kozlov et al. 2019) and the GTR+GAMMA model of evolution. After confirming that all copies clustered together on the LSU gene tree (supplementary fig. 6), the longest sequence was selected as a representative to be included in the concatenated dataset alongside other single-copy markers. Trimmed single-copy gene alignments were concatenated using AMAS v0.98 (Borowiec 2016) and the concatenated alignment for each lineage was run in RAxML-NG with genes partitioned and the GTR+GAMMA model of evolution.

All results were plotted in R v4.1.1 using the following packages: ape v5.5 (Paradis and Schliep 2019), ggplot2 v3.3.5 (Wickham 2016), ggpubr v0.4.0 (Kassambara 2020), ggtree v3.0.4 (Yu et al. 2012), and tidyverse v1.3.2 (Wickham 2019). R scripts were written using RStudio v2021.09.1+372 (RStudio Team 2015). This research utilised Queen Mary’s Apocrita HPC facility, supported by QMUL Research-IT (Butcher et al. 2017). Scripts of all analyses are available at https://github.com/Rowena-h/EndophyteGenomes.

## Supplementary Material

evad038_Supplementary_Data

## Data Availability

WGS data and annotated genome assemblies are available on GenBank under the BioProject accession PRJNA786750. Scripts of all analyses are available at https://github.com/Rowena-h/EndophyteGenomes.
