# Supplementary material for: Tapping Culture Collections for Fungal Endophytes: First Genome Assemblies for Three Genera and Five Species in the Ascomycota
Source: Genome Biol Evol. 2023 Mar 7;15(3):evad038. doi: 10.1093/gbe/evad038 (PMC10027605; doi:10.1093/gbe/evad038)
Supplement: evad038_Supplementary_Data [file evad038_supplementary_data.pdf]

Supplementary Material

Tapping culture collections for fungal endophytes: first genome assemblies for three genera and five species in the *Ascomycota*

Rowena Hill, Quentin Levicky, Frances Pitsillides, Amy Junnonen, Elena Arrigoni, J. Miguel Bonnin, Anthony Kermode, Sahr Mian, Ilia J. Leitch, Alan G. Buddie, Richard J. A. Buggs and Ester Gaya

Taxa used for protein and EST evidence during annotation

Files for the following taxa were downloaded from MycoCosm (Grigoriev et al., 2014):

Gnomoniopsis castanea Behrend (Gnocas1) for IMI 355080 and IMI 355082 (unpublished); *Colletotrichum somersetensis* CBS 131599 (Colso1) for IMI 355084 and IMI 366226 (Baroncelli et al., 2022); *Didymella exigua* CBS 182.55 (Didex1) for IMI 355091, IMI 355093, IMI 359910, IMI 360193, and IMI 364377 (Haridas et al., 2020); *Pyrenochaeta* sp. MPI-SDFR-AT-0127 (Pyrly1) for IMI 356814 (Mesny et al., 2021); *Bimuria novae-zelandiae* CBS 107.79 (Bimnz1) for IMI 356815 and IMI 367209 (Haridas et al., 2020); *Neurospora crassa* 73 trp-3 (Neucr_trp3_1) for IMI 360204 (Baker et al., 2015); *Chaetomium globosum* MPI-SDFR-AT-0079 (Chagl1) for IMI 366227 (Mesny et al., 2021); and *Fusarium solani* FSSC 5 MPI-SDFR-AT-0091 (Fusso1) for IMI 366586 (Mesny et al., 2021).

Cytometric genome size estimation methods

Two different fungal strains were used as internal calibration standards to estimate the genome sizes of the endophyte strains. The first internal fungal standard was a strain of *Coprinellus micaceus* which had been isolated and cultured from a collection made by R. Wright on 05/10/2020 at Royal Botanic Gardens Kew, UK (culture code: FTOL_0141). The genome size of *C. micaceus* was estimated directly by co-running a sample with *Arabidopsis thaliana* (L.) Heynh., 1842 (ecotype col-0 NASC) with an estimated genome size of 172.44 Mbp/1C. *C. micaceus* mycelium was co-chopped with 1 cm^2 fresh *A. thaliana* leaf tissue in a petri dish with 1 ml of LB01 buffer (Doležel, Binarová, and Lucretti, 1989). A further 1 ml of LB01 was added to the sample and the contents gently mixed. The sample was then passed through a 30 µm nylon filter, stained with 100 µl propidium iodide (1 mg/ml) and incubated on ice for 10 minutes before running through a Sysmex CyFlow Space flow cytometer (Sysmex Partec GmbH, Görlitz, Germany) fitted with a 100 mW green solid state laser (532 nm, Cobolt Samba, Solna, Sweden). Each isolate was run through the flow cytometer three times to ensure reproducibility of results, with at least 1,000 nuclei analysed each time. Once the genome size of *C. micaceus* had been estimated (62.62 Mbp/1C) it was then used to calibrate a second internal standard, *Coprinopsis piacea* (52.83 Mbp/1C), which was isolated and cultured from a collection that had been made by R. Wright on 17/12/2020 at Royal Botanic Gardens Kew, UK (culture code: FTOL_0189). Preparation of each

endophyte sample for flow cytometry was then completed following the same process as above, except using one of the two internal fungal standards instead of *A. thaliana*.

We used the Partec FloMax v2.4d software (Sysmex Partec GmbH) to produce histograms showing the relative fluorescence of nuclei (Supplementary Figure 5). FlowMax gating tools were used to generate linear regressions to gate nuclei and quantify the number of nuclei and coefficient of variation (CV) of each peak. A polygonal region was drawn around the nuclei in the side scatter cytogram to improve the quality of the peaks by ensuring only intact nuclei were analysed. The measurement of DNA content for each isolate was considered reliable only if the CV value of the G_1 peak was below the accepted limit of 10% for fungi (Bourne et al., 2014). The holoploid 1C genome size of each strain was estimated using the following formula:

$$\frac{\text{Mean } G_1 \text{ fluorescence peak of sample} \times \text{1C nuclear DNA content of reference standard}}{\text{Mean } G_1 \text{ fluorescence peak of reference standard}}$$

Genome size in Mbp was calculated using the conversion factor 1 pg = 978 Mbp (Doležel, Bartoš, et al., 2003).

Supplementary Table 1: Assembly statistics from all assembly tools for the 15 endophyte strains.

			QUAST						BUSCO
			# contigs ≥500bp	Largest contig (bp)	Total length (bp)	GC (%)	N50	L50	Single-copy BUSCOs (Completeness)
Short-read	355080	ABySS k72	1,521	331,203	40,667,570	51.60	60,408	212	1,542 (90.39%)
		MEGAHIT	1,832	202,743	40,947,610	51.59	52,207	247	1,647 (96.54%)
		SPAdes	798	564,097	41,065,971	51.57	126,266	102	1,653 (96.89%)
	355091	ABySS k80	1,184	576,573	34,975,890	53.45	132,824	81	1,682 (98.59%)
		MEGAHIT	1,526	421,407	34,840,787	53.46	103,515	101	1,683 (98.65%)
		SPAdes	610	908,435	35,454,550	53.43	214,263	44	1,688 (98.94%)
	359910	ABySS k64	1,937	202,055	33,882,229	52.73	42,393	244	1,643 (96.31%)
		MEGAHIT	2,408	173,198	34,289,510	52.52	40,722	261	1,651 (96.78%)
		SPAdes	1,357	259,290	34,499,516	52.37	73,383	152	1,667 (97.71%)
	360193	ABySS k88	1,356	476,436	35,335,285	53.37	85,694	121	1,672 (98.01%)
		MEGAHIT	1,719	325,895	35,071,700	53.46	74,746	139	1,681 (98.53%)
		SPAdes	776	641,373	35,521,025	53.41	178,807	56	1,690 (99.06%)
	360204	ABySS k72	4,475	120,442	36,976,442	52.58	16,432	622	1,567 (91.85%)
		MEGAHIT	6,029	107,474	37,710,625	52.61	13,214	809	1,604 (94.02%)
		SPAdes	4,925	166,179	45,121,419	52.54	24,778	433	1,636 (95.90%)
	364337	ABySS k72	1,734	268,349	30,277,320	51.69	50,318	178	1,643 (96.31%)
		MEGAHIT	2,116	237,638	30,535,934	51.48	45,506	201	1,663 (97.48%)
		SPAdes	1,155	382,275	30,512,631	51.44	74,080	124	1,676 (98.24%)
	366226	ABySS k64	2,708	189,189	54,213,268	53.63	39,154	421	1,627 (95.37%)
		MEGAHIT	3,295	193,772	54,922,345	53.62	34,099	481	1,628 (95.43%)
		SPAdes	1,830	305,111	55,288,883	53.54	63,290	275	1,655 (97.01%)
	366586	ABySS k72	1,656	320,053	40,977,177	52.57	50,683	239	1,657 (97.13%)
		MEGAHIT	2,221	205,757	41,829,246	52.43	42,500	299	1,663 (97.48%)
		SPAdes	1,411	470,694	42,358,263	52.22	90,196	139	1,669 (97.83%)
Hybrid	355082	Flye	12	7,084,357	40,197,374	50.70	6,429,383	3	1,668 (97.77%)
		Raven	15	7,080,637	40,228,030	50.67	4,326,196	4	1,667 (97.71%)
		hybridSPAdes	281	1,693,788	39,888,836	51.10	413,748	29	1,647 (96.54%)
	355084	Flye	58	7,342,820	49,508,467	51.90	2,983,733	6	1,643 (96.31%)
		Raven	56	3,110,953	49,524,676	51.84	1,317,902	13	1,664 (97.54%)
		hybridSPAdes	753	840,111	49,421,028	52.47	161,131	88	1,683 (98.65%)
	355093	Flye	86	2,369,202	31,358,738	52.97	1,219,652	10	1,687 (98.89%)
		Raven	27	1,884,042	31,528,740	52.85	1,301,886	10	1,684 (98.71%)
		hybridSPAdes	184	1,552,342	31,829,418	52.87	520,122	18	1,687 (98.89%)
	356814	Flye	89	3,269,191	34,410,298	50.41	1,599,529	8	1,677 (98.30%)
		Raven	24	2,991,912	34,846,001	50.24	1,616,366	9	1,678 (98.36%)
		hybridSPAdes	593	698,129	33,512,421	51.19	148,079	67	1,673 (98.07%)
	356815	Flye	54	5,272,851	38,910,400	51.57	4,473,122	4	1,680 (98.48%)
		Raven	11	5,345,287	39,450,705	51.25	4,705,368	4	1,672 (98.01%)
		hybridSPAdes	362	1,812,647	38,868,017	51.75	485,797	25	1,677 (98.30%)
	366227	Flye	162	3,665,392	30,332,852	55.81	962,134	9	1,604 (94.02%)
		Raven	52	2,828,572	29,586,632	55.79	1,760,284	7	1,499 (87.87%)
		hybridSPAdes	2,530	149,393	29,037,354	55.70	19,002	435	1,522 (89.21%)
	367209	Flye	97	4,017,923	42,713,253	49.79	1,630,038	9	1,656 (97.07%)
		Raven	31	4,380,344	42,809,244	49.69	2,200,773	7	1,675 (98.18%)
		hybridSPAdes	684	1,149,365	42,184,608	50.32	323,849	40	1,689 (99.00%)

Supplementary Table 2: Flow cytometry genome size estimation results. * = *Coprinopsis piacea*, † = *Coprinellus micaceous*. Cytometric completeness = (assembly length (Mbp) / genome size (Mbp/1C)) × 100.

IMI	Mean G_1 peak		Mean CV		Genome size (pg/1C)	Genome size (Mbp/1C)	Cytometric completeness (%)
	Sample	Standard	Sample	Standard			
355093	150.09	242.92*	7.26	3.59*	0.033	32.03	98.44
356814	241.55	341.96†	5.03	3.42†	0.045	44.21	77.08
359910	233.33	346.14†	4.98	5.20†	0.043	42.19	77.92
360204	324.06	412.41†	4.16	5.07†	0.050	49.18	75.09
364377	175.70	412.41†	6.29	5.55†	0.040	39.55	75.95

Supplementary Table 3: Metadata for the endophyte strains studied here.

IMI	CABI name	Updated name	Taxonomy	Host	Origin
355080	*Phomopsis*	*Gnomoniopsis* sp.	*Gnomoniaceae*, *Diaporthales*, *Sordariomycetes*	*Quercus ilex*	Lugano, Switzerland
355082	*Phomopsis*	*Gnomoniopsis smithogilvyi*	*Gnomoniaceae*, *Diaporthales*, *Sordariomycetes*	*Quercus ilex*	Lugano, Switzerland
355084	*Colletotrichum acutatum*	*Colletotrichum fioriniae*	*Glomerellaceae*, *Glomerellales*, *Sordariomycetes*	*Quercus ilex*	Lugano, Switzerland
355091	*Phoma sorghina*	*Didymella pomorum*	*Didymellaceae*, *Pleosporales*, *Dothideomycetes*	*Opuntia* sp.	Queensland, Australia
355093	*Phoma*	*Didymella* sp.	*Didymellaceae*, *Pleosporales*, *Dothideomycetes*	*Opuntia* sp.	Queensland, Australia
356814	*Phoma leveillei*	*Neocucurbitaria cava*	*Cucurbitariaceae*, *Pleosporales*, *Dothideomycetes*	*Quercus ilex*	Mallorca, Spain
356815	*Leptosphaeria coniothyrium*	*Didymosphaeria variabile*	*Didymosphaeriaceae*, *Pleosporales*, *Dothideomycetes*	*Quercus ilex*	Mallorca, Spain
359910	*Phoma*	*Ascochyta clinopodiicola*	*Didymellaceae*, *Pleosporales*, *Dothideomycetes*	*Dryas octopetala*	Switzerland
360193	*Microsphaeropsis*	*Didymella glomerata*	*Didymellaceae*, *Pleosporales*, *Dothideomycetes*	*Gynoxis oleifolia*	Ecuador
360204	*Gelasinospora*	*Neurospora* sp.	*Sordariaceae*, *Sordariales*, *Sordariomycetes*	*Gynoxis oleifolia*	Ecuador
364377	*Phoma nebulosa*	*Neodidymelliopsis* sp.	*Didymellaceae*, *Pleosporales*, *Dothideomycetes*	*Persea americana*[1]	Trinidad and Tobago
366226	*Colletotrichum crassipes*	*Colletotrichum tropicale*	*Glomerellaceae*, *Glomerellales*, *Sordariomycetes*	*Manilkara bidentata*	Puerto Rico
366227	*Colletotrichum crassipes*	*Collariella* sp.	*Chaetomiaceae*, *Sordariales*, *Sordariomycetes*	*Manilkara bidentata*	USA[2]
366586	*Fusarium solani*	*Neocosmospora piperis*	*Nectriaceae*, *Hypocreales*, *Sordariomycetes*	*Manilkara bidentata*	Puerto Rico
367209	*Leptosphaeria coniothyrium*	cf. *Kalmusia* sp.	*Didymosphaeriaceae*, *Pleosporales*, *Dothideomycetes*	*Manilkara bidentata*	Puerto Rico

[1]Isolated as endophyte of leaves imported by leafcutter ants into their nests.
[2]Suspected input error based on adjacent IMI records.

Supplementary Table 4: GenBank accession numbers for taxa used in the phylogenetic analyses. ^{T} = ex-type, ^{ET} = ex-epitype. *Ascochyta* sampling informed by Hou et al. (2020).

	Name	Voucher	RPB2	TUB2
***Ascochyta* (Figure 3B)**	*Ascochyta astragalina*	CBS 113797 = UPSC 2222	MT018257	KT389776
	A. benningiorum	CBS 144957^{T} = JW 196005	MN824606	MN824755
	A. benningiorum	JW 196013	MN824608	MN824757
	A. benningiorum	JW 196023	MN824607	MN824756
	A. clinopodiicola	CBS 123524		MT005693
	A. clinopodiicola	CBS 123527		MT005694
	A. clinopodiicola	CBS 123526		MT005692
	A. clinopodiicola	CBS 127776		MT005695
	A. coronillae-emeri	MFLUCC 13-0820^{T}	MH069679	MH069686
	A. fabae	CBS 524.77	MT018241	GU237526
	A. herbicola	CBS 629.97 = PD 76/1017	KP330421	GU237614
	A. koolunga	CBS 189.91	MN983286	MN983711
	A. koolunga	DAR 78535^{T}	EU874849	
	A. lentis	CBS 231.79 = DAOM 170658	MT018248	MT005689
	A. medicaginicola	CBS 112.53^{T}	MT018251	GU237628
	A. nigripycnidia	CBS 116.96^{T} = PD 95/7930	MT018253	GU237637
	A. phacae	CBS 184.55^{T}	MT018255	KT389769
	A. pilosella	CBS 583.97^{T}	MT018258	MT005696
	A. pisi	CBS 122785^{T} = PD 78/517	MT018244	GU237532
	A. rabiei	CBS 237.37^{T}	MT018256	KT389773
	A. rosae	MFLUCC 15-0063^{T}	KY514409	
	A. sp.	CBS 136887	MN983295	KX033387
	A. syringae	CBS 126.82	MN983308	MN983728
	A. viciae	CBS 451.68	KT389562	KT389778
	A. viciae-pannonicae	CBS 254.92	MT018250	KT389779
	A. viciae-villosae	CBS 255.92	MT018249	MT005690
	Phoma herbarum	CBS 615.75 = IMI 199779 = PD	KP330420	FJ427133

Supplementary Table 4 continued.

Name	Voucher	RPB2	TUB2
Phomatodes aubrietiae	CBS 627.97^{T} = PD 70/714	KT389665	GU237585

Supplementary Table 4 continued. *Collariella* sampling informed by Wang, Houbraken, et al. (2016) and Wang, Han, et al. (2022).

	Name	Voucher	RPB2	TUB2
***Collariella* (Figure 3H)**	*Achaetomium globosum*	CBS 332.67[T]	KX976793	KX976911
	Melanocarpus albomyces	CBS 638.94[T]	KX976886	KX977021
	Ovatospora brasiliensis	CBS 130174	KX976895	KX977030
	Collariella bostrychodes	CBS 163.73	KX976837	KX976983
	Colla. bostrychodes	CBS 586.83	KX976838	KX976984
	Colla. bostrychodes	DTO 319-C4		KX976985
	Colla. bostrychodes	DTO 324-H3 = DTO 324-H6	KX976839	KX976986
	Colla. bostrychodes	CBS 121706		KX976987
	Colla. causiiformis	CBS 792.83[T]	KX976840	KX976988
	Colla. carteri	CBS 128.85[T]	KX976841	KX976989
	Colla. gracilis	CBS 146.60[T]	KX976842	KX976990
	Colla. gracilis	CBS 249.75	KX976843	KX976991
	Colla. quadrangulata	CBS 142.58	KX976844	KX976992
	Colla. quadrangulata	CBS 152.59	KX976845	KX976993
	Colla. robusta	CBS 551.83[T]	KX976846	KX976994
	Colla. robusta	CBS 508.84	KX976847	KX976995
	Colla. virescens	CBS 148.68[T]	KX976848	KX976996
	Colla. virescens	CBS 547.75	KX976849	KX976997
	Colla. anguipilia	CBS 632.83	MZ342989	MZ343028
	Colla. quadrum	CGMCC:3.17920 = LC5782	KY575873	KU746770
	Colla. quadrum	CGMCC:3.17919 = LC5781	KY575872	KU746769
	Colla. quadrum	CGMCC:3.17918 = LC5693	KY575871	KU746768
	Colla. quadrum	CGMCC:3.17917 = LC5446	KY575870	KU746767
	Colla. hexagonospora	CBS 171.84	MZ342977	MZ343016
	Colla. pachypodioides	CBS 164.52	MZ342975	MZ343014
	Colla. carteri	SCUA-Saf-O26	MW671060	MW671081
	Colla. carteri	HGUP191086		MZ724096
	Colla. carteri	D32	MG890121	

Supplementary Table 4 continued.

Name	Voucher	RPB2	TUB2
Colla. carteri	ChL-A48		MG890023
Colla. sp.	SCUA-Agh-20H	MN520427	MN520423
Colla. sp.	SCUA-Agh-20H-2	MN520426	MN520422

Supplementary Table 4 continued. *Colletotrichum* sampling informed by Vieira et al. (2020) and Liu et al. (2022).

	Name	Voucher	GAPDH	HIS3	TUB2
***Colletotrichum* (Figure 3G)**	*Colletotrichum abscissum*	COAD 1877^{T}	KP843129	KP843138	KP843135
	Colle. acerbum	CBS 128530 = ICMP 12921 = PRJ 1199.3^{T}	JQ948790	JQ949450	JQ950110
	Colle. acidae	MFLUCC 17–2659^{T}	MH003691		MH003700
	Colle. acutatum	CBS 127545 = CPC 13947	JQ948714	JQ949374	JQ950034
	Colle. acutatum	CBS 112996 = ATCC 56816 = STE-U 5292^{T}	JQ948677	JQ005818	JQ005860
	Colle. aenigma	ICMP 18608^{T}	JX010044		JX010389
	Colle. aeschynomenes	ICMP 17673^{T} = ATCC 201874	JX009930		JX010392
	Colle. alatae	CBS 304.67^{T} = ICMP 17919	JX009990		JX010383
	Colle. alienum	ICMP 12071^{T}	JX010028		JX010411
	Colle. americae-borealis	CBS 136232^{T}	KM105579	KM105364	KM105504
	Colle. annellatum	CBS 129826 = CH1^{T}	JQ005309	JQ005483	JQ005656
	Colle. anthrisci	CBS 125334^{T}	GU228237	GU228041	GU228139
	Colle. antirrhinicola	CBS 102189^{T}	KM105531	KM105320	KM105460
	Colle. aotearoa	ICMP 18537^{T}	JX010005		JX010420
	Colle. arecicola	CGMCC 3.19667^{T}	MK935455		MK935498
	Colle. artocarpicola	MFLUCC 18-1167^{T}	MN435568		MN435567
	Colle. arxii	CBS 132511^{T}	KF687843	KF687858	KF687881
	Colle. asianum	ICMP 18580^{T} = CBS 130418	JX010053		JX010406
	Colle. australe	CBS 116478 = HKUCC 2616^{T}	JQ948786	JQ949446	JQ950106
	Colle. australianum	VPRI 43075^{T}	MG572127		MG572149
	Colle. beeveri	CBS 128527 = ICMP 18594^{T}	JQ005258	JQ005432	JQ005605
	Colle. bidentis	COAD 1020^{T} = CPC 21930	KF178506	KF178554	KF178602
	Colle. bletillum	CGMCC 3.15117^{T}	KC843506		JX625207
	Colle. boninense	CBS 123755 = MAFF 305972^{T}	JQ005240	JQ005414	JQ005588
	Colle. brasiliense	CBS 128501 = ICMP 18607 = PAS12^{T}	JQ005322	JQ005496	JQ005669
	Colle. brassicicola	CBS 101059 = LYN 16331^{T}	JQ005259	JQ005433	JQ005606
	Colle. brevisporum	CBS 129957	MG600822	MG600908	MG601029
	Colle. brisbanense	CBS 292.67 = DPI 11711^{T}	JQ948621	JQ949282	JQ949942

Supplementary Table 4 continued. *Colletotrichum* sampling informed by Vieira et al. (2020) and Liu et al. (2022)

	Name	Voucher	GAPDH	HIS3	TUB2
***Colletotrichum* (Figure 3G)**	*Colle. bryoniicola*	CBS 109849^T	KM105532	KM105321	KM105461
	Colle. cacao	CBS 119297^T	MG600832	MG600916	MG601039
	Colle. cairnsense	RIP 63642^T = CBS 140847	KU923704	KU923722	KU923688
	Colle. camelliae	CGMCC 3.14925 = LC1364^T	KJ954782	MZ673847	KJ955230
	Colle. catinaense	CBS 142417 = CPC 27978	KY856224	KY856307	KY856482
	Colle. catinaense	CBS 142416 = CPC 28019	KY856223	KY856306	KY856481
	Colle. cattleyicola	CBS 170.49^T	MG600819	MG600905	MG601025
	Colle. cereale	CBS 129663 = KS20BIG			JQ005858
	Colle. changpingense	CGMCC 3.17582^T = SA0016 = MFLUCC 15-0022	MZ664048		MZ673952
	Colle. chiangmaiense	MFLUCC 18-0945^T	MW548592		
	Colle. chrysanthemi	IMI 364540 = CPC 18930^T	JQ948603	JQ949264	JQ949924
	Colle. chrysophilum	CMM4268^T	KX094183		KX094285
	Colle. circinans	CBS 221.81^T	GU228247	GU228051	GU228149
	Colle. clidemiae	ICMP 18658^T	JX009989		JX010438
	Colle. clivicola	CBS 125375^T	MG600795	MG600892	MG601000
	Colle. cobbittiense	BRIP 66219^T	MH094133	MH094136	MH094137
	Colle. coelogynes	CBS 132504^T	MG600776	MG600882	MG600980
	Colle. colombiense	CBS 129818 = G2^T	JQ005261	JQ005435	JQ005608
	Colle. conoides	CGMCC 3.17615 = CAUG17 = LC6226^T	KP890162		KP890174
	Colle. constrictum	CBS 128504 = ICMP 12941^T	JQ005325	JQ005499	JQ005672
	Colle. corchorum-capsularis	FAFU 03	KT439361		KT439341
	Colle. cordylinicola	MFLUCC 090551^T = ICMP 18579	JX009975		JX010440
	Colle. cosmi	CBS 853.73 = PD 73/856^T	JQ948604	JQ949265	JQ949925
	Colle. costaricense	CBS 330.75^T	JQ948510	JQ949171	JQ949831
	Colle. curcucmae	IMI 288937^T	GU228285		GU228187
	Colle. cuscutae	IMI 304802 = CPC 18873^T	JQ948525	JQ949186	JQ949846
	Colle. cymbidicola	IMI 347923^T	JQ005253	JQ005427	JQ005600
	Colle. dacrycarpi	CBS 130241 = ICMP 19107^T	JQ005323	JQ005497	JQ005670

Supplementary Table 4 continued. *Colletotrichum* sampling informed by Vieira et al. (2020) and Liu et al. (2022)

	Name	Voucher	GAPDH	HIS3	TUB2
***Colletotrichum* (Figure 3G)**	*Colle. dematium*	CBS 125.25^{T}	GU228211	GU228015	GU228113
	Colle. destructivum	CBS 136228^{T}	KM105561	KM105347	KM105487
	Colle. destructivum	CBS 136852	KM105562	KM105348	KM105488
	Colle. dracaenophilum	CBS 118199^{T}	JX546707	JX546756	JX519247
	Colle. endophyticum	MFLUCC 13-0418 = LC0324^{T}	KC832854	MZ673839	MZ673954
	Colle. eremochloae	CBS 129661^{T} = C05			JX519245
	Colle. falcatum	CGMCC 3.14187 = CBS 147945^{T}			JQ005856
	Colle. fioriniae	CBS 293.67,DPI 13120	JQ948640	JQ949301	JQ949961
	Colle. fioriniae	CBS 128517 = ARSEF 10222 = ERL 1257 = EHS 58^{T}	JQ948622	JQ949283	JQ949943
	Colle. fioriniae	CBS 129948	JQ948674	JQ949335	JQ949995
	Colle. fioriniae	CBS 119293	JQ948644	JQ949305	JQ949965
	Colle. fructi	CBS 346.37 / CCT 4806^{T}	GU228236	GU228040	GU228138
	Colle. fructicola	1087	KX094174		KX094279
	Colle. fructicola	3589	KX094175		KX094280
	Colle. fuscum	CBS 133701^{T}	KM105524	KM105314	KM105454
	Colle. fusiforme	MFLUCC 12–0437T	KT290255		KT290256
	Colle. gigasporum	CBS 101881	KF687841	KF687861	KF687886
	Colle. gloeosporioides	IMI 356878^{T} = ICMP 17821 = CBS 112999	JX010056	JQ005413	JX010445
	Colle. godetiae	CBS 133.44^{T}	JQ948733	JQ949393	JQ950053
	Colle. graminicola	CBS 130836^{T} M1001			JQ005851
	Colle. grevilleae	CBS 132879 = CPC 15481	KC297010	KC297056	KC297102
	Colle. grossum	CGMCC3.17614 = CAUG7 = LC6227^{T}	KP890159		KP890171
	Colle. guajave	IMI 350839^{T}	JQ948600	JQ949261	JQ949921
	Colle. guizhouensis	CGMCC 3.15112^{T}	KC843507		JX625185
	Colle. hebeiense	MFLUCC13–0726^{T}	KF377495		KF288975
	Colle. hederiicola	CBS 142418 = CPC 26844^{T}	KY856270	KY856361	KY856528
	Colle. henanense	LC3030 = CGMCC 3.17354 = LF238 ^{T}	KJ954810	MZ673835	KJ955257

Supplementary Table 4 continued. *Colletotrichum* sampling informed by Vieira et al. (2020) and Liu et al. (2022)

	Name	Voucher	GAPDH	HIS3	TUB2
***Colletotrichum* (Figure 3G)**	*Colle. higginsianum*	IMI 349061 = CPC 19379^{T}	KM105535	KM105324	KM105464
	Colle. hippeastri	CBS 125376 = CSSG1^{T}	JQ005318	JQ005492	JQ005665
	Colle. horii	NBRC 7478^{T} = ICMP 10492 = MTCC 10841	GQ329681		JX010450
	Colle. hystricis	CBS 142411 = CPC 28153^{T}	KY856274	KY856365	KY856532
	Colle. incanum	ATCC 64682^{T}	KC110807		KC110816
	Colle. indonesiense	CBS 127551 = CPC 14986^{T}	JQ948618	JQ949279	JQ949939
	Colle. jiangxiense	CGMCC 3.17361^{T} = LC3266 = LF488	KJ954850		OK236389
	Colle. johnstonii	CBS 128532 = ICMP 12926 = PRJ 1139.3^{T}	JQ948775	JQ949435	JQ950095
	Colle. kahawae	IMI 319418^{T} = ICMP 17816	JX010012	MZ673838	JX010444
	Colle. karstii	CBS 111998	JQ005299	JQ005473	JQ005646
	Colle. kinghornii	CBS 198.35^{T}	JQ948785	JQ949445	JQ950105
	Colle. laticiphilum	CBS 112989 = IMI 383015 = STE-U 5303^{T}	JQ948619	JQ949280	JQ949940
	Colle. lentis	CBS 127604 = DAOM 235316 = CT21^{T}	KM105597	JQ005808	JQ005850
	Colle. lilii	CBS 109214	GU228202		GU228104
	Colle. limetticola	CBS 114.14^{T}	JQ948523	JQ949184	JQ949844
	Colle. limonicola	CBS 142410 = CPC 31141	KY856296	KY856388	KY856554
	Colle. lindemuthianum	CBS 144.31^{T}	JX546712	JQ005821	JQ005863
	Colle. lineola	CBS 125337^{T}	GU228221	GU228025	GU228123
	Colle. lini	CBS 172.51^{T}	KM105581	JQ005807	JQ005849
	Colle. liriopes	CBS 119444^{T}	GU228196		GU228098
	Colle. lobatum	IMI 79736^{T}	MG600828	MG600912	MG601035
	Colle. lupini	CBS 109225 = BBA 70884^{T}	JQ948485	JQ949146	JQ949806
	Colle. magnum	CBS 519.97^{T}	MG600829	MG600913	MG601036
	Colle. makassarense	CBS 143664^{T}	MH728820		MH846563
	Colle. malvarum	CBS 521.97^{T} = LARS 720 = Lav-4	KF178504	KF178553	KF178601
	Colle. melonis	CBS 159.84^{T}	JQ948524	JQ949185	JQ949845
	Colle. merremiae	CBS 124955^{T}	MG600825	MG600910	MG601032
	Colle. musae	CMM4422	KX094189		KX094298

Supplementary Table 4 continued. *Colletotrichum* sampling informed by Vieira et al. (2020) and Liu et al. (2022)

	Name	Voucher	GAPDH	HIS3	TUB2
***Colletotrichum* (Figure 3G)**	*Colle. musae*	CMM4423	KX094195		KX094294
	Colle. musae	CMM4445	KX094188		KX094293
	Colle. musicola	CBS 132885^T	MG600798	MG600895	MG601003
	Colle. navitas	CBS 125086^T			JQ005853
	Colle. novae-zelandiae	CBS 128505 = ICMP 12944^T	JQ005315	JQ005489	JQ005662
	Colle. nupharicola	CBS 470.96^T = ICMP 18187	JX009972		JX010398
	Colle. nymphaeae	CBS 515.78^T	JQ948527	JQ949188	JQ949848
	Colle. ocimi	CBS 298.94^T	KM105577	KM105362	KM105502
	Colle. oncidii	CBS 129828^T	JQ005256	JQ005430	JQ005603
	Colle. orbiculare	CBS 570.97^T = LARS 73	KF178490	KF178539	KF178587
	Colle. orchidearum	CBS 135131^T	MG600800	MG600897	MG601005
	Colle. panamense	CBS 125386^T	MG600826	MG600911	MG601033
	Colle. pandanicola	MFLUCC 17-0571^T	MG646934		MG646926
	Colle. paranaense	CBS 134729 = Col 19 = CPC 20901^T	KC205026	KC205004	KC205060
	Colle. parsonsiae	CBS 128525 = ICMP 18590^T	JQ005320	JQ005494	JQ005667
	Colle. paxtonii	IMI 165753 = CPC 18868 ^{T}	JQ948615	JQ949276	JQ949936
	Colle. perseae	CBS 141365^T = GA100	KX620242		KX620341
	Colle. petchii	CBS 378.94^T	JQ005310	JQ005484	JQ005657
	Colle. phormii	CBS 118194 = AR 3546^T	JQ948777	JQ949437	JQ950097
	Colle. phyllanthi	CBS 175.67 = MACS 271^T	JQ005308	JQ005482	JQ005655
	Colle. piperis	CPC 21195^T	MG600820	MG600906	MG601027
	Colle. pisicola	CBS 724.97 = LARS 60^T	KM105522	KM105312	KM105452
	Colle. plurivorum	CBS 125474^T	MG600781	MG600887	MG600985
	Colle. proteae	CBS 132882^T = CPC 14859	KC297009	KC297045	KC297101
	Colle. pseudomajus	CBS 571.88^T	KF687826	KF687864	KF687883
	Colle. pseudotheobromicola	MFLUCC 18–1602^T	MH853675		MH853684
	Colle. psidii	CBS 145.29^T = ICMP 19120	JX009967		JX010443
	Colle. pyricola	CBS 128531 = ICMP 12924 = PRJ 977.1^T	JQ948776	JQ949436	JQ950096

Supplementary Table 4 continued. *Colletotrichum* sampling informed by Vieira et al. (2020) and Liu et al. (2022)

	Name	Voucher	GAPDH	HIS3	TUB2
***Colletotrichum* (Figure 3G)**	*Colle. queenslandicum*	CMM3233	MF110849		MF111058
	Colle. queenslandicum	CMM3241	MF110848		MF111059
	Colle. queenslandicum	CMM3236	MF110850		MF111060
	Colle. radicis	CBS 529.93^{T}	KF687825	KF687847	KF687869
	Colle. rhexiae	Coll1026 = BPI 884112 = CBS 133134^{T}	MZ664046	MZ673834	JX145179
	Colle. rhombiforme	CBS 129953 = PT250 = RB011^{T}	JQ948788	JQ949448	JQ950108
	Colle. riograndense	ICMP 20083^{T}	KM655298		KM655300
	Colle. salicis	CBS 607.94^{T}	JQ948791	JQ949451	JQ950111
	Colle. salsolae	ICMP 19051^{T}	JX009916		JX010403
	Colle. scovillei	CBS 126529 = PD 94/921-3 = BBA 70349^{T}	JQ948597	JQ949258	JQ949918
	Colle. siamense	CBS133123	KX094186		KX094289
	Colle. sidae	CBS 504.97^{T}	KF178497	KF178545	KF178593
	Colle. simmondsii	BRIP 28519 = CBS 122122^{T}	JQ948606	JQ949267	JQ949927
	Colle. sloanei	IMI 364297 = CPC 18929^{T}	JQ948617	JQ949278	JQ949938
	Colle. sojae	ATCC 62257^{T}	MG600810	MG600899	MG601016
	Colle. spaethianum	CBS 167.49^{T}	GU228199		GU228101
	Colle. spinaceae	CBS 128.57	GU228239	GU228043	GU228141
	Colle. spinosum	CBS 515.97^{T} = LARS 465 = DAR 48942	KF178498	KF178547	KF178595
	Colle. sublineola	CBS 131301^{T} = S3.001			JQ005855
	Colle. tabacum	N150 = CPC 18945^{T}	KM105557	KM105344	KM105484
	Colle. tainanense	CBS 143666^{T}	MH728823		MH846558
	Colle. tamarilloi	CBS 129814 = T.A.6^{T}	JQ948514	JQ949175	JQ949835
	Colle. tebeestii	CBS 522.97^{T} = LARS 733 = 83-43	KF178505	KF178546	KF178594
	Colle. temperatum	CBS 133122^{T} = Coll883 = BPI 884100	MZ664045	MZ673833	JX145211
	Colle. theobromicola	CMM4242	KX094173		KX094278
	Colle. theobromicola	CMM3214	MF110847		MF111049
	Colle. theobromicola	CMM3221	MF110855		MF111048
	Colle. tofieldiae	CBS 495.85	GU228193		GU228095

Supplementary Table 4 continued. *Colletotrichum* sampling informed by Vieira et al. (2020) and Liu et al. (2022)

	Name	Voucher	GAPDH	HIS3	TUB2
***Colletotrichum* (Figure 3G)**	*Colle. torulosum*	CBS 128544 = ICMP 18586^{T}	JQ005251	JQ005425	JQ005598
	Colle. trifolii	CBS 158.83^{T}	KF178502	KF178551	KF178599
	Colle. tropicale	CMM4243	KU213601		KU213604
	Colle. tropicale	CMM2999	MF110846		MF111088
	Colle. tropicicola	CBS 127555	MG600778	MG600884	MG600982
	Colle. truncatum	CBS 151.35^{T}	GU228254		GU228156
	Colle. utrechtense	CBS 130243^{T}	KM105554	KM105341	KM105481
	Colle. verruculosum	IMI 45525^{T}	GU228198		GU228100
	Colle. vietnamense	CBS 125478^{T}	KF687832	KF687855	KF687877
	Colle. vignae	CBS 501.97 = LARS 56^{T}	KM105534	KM105323	KM105463
	Colle. viniferum	GZAAS 5.08601^{T} = yg1	JN412798		
	Colle. vittalense	CBS 181.82^{T}	MG600796	MG600893	MG601001
	Colle. walleri	CBS 125472 = BMT(HL)19^{T}	JQ948605	JQ949266	JQ949926
	Colle. wuxiense	CGMCC 3.17894^{T}	KU252045		KU252200
	Colle. xanthorrhoeae	BRIP 45094^{T} = ICMP 17903 = CBS 127831	JX009927		JX010448
	Colle. xishuangbannaense	MFLUCC 19-0107^{T}	MW537586		
	Colle. yulongense	CFCC 50818^{T}	MK108986		MK108987
	Colle. yunnanense	CBS 132135	JX546706	JX546755	JX519248
	Monilochaetes infuscans	CBS 869.96	JX546612	JQ005822	JQ005864

Supplementary Table 4 continued. *Didymella* sampling informed by Chen et al. (2017) and Scarpari et al. (2020).

	Name	Voucher	RPB2	TUB2
***Didymella* (Figure 3A)**	*Didymella acetosellae*	CBS 179.97	KP330415	GU237575
	D. aeria	LC 8120	KY742138	KY742294
	D. aeria	CGMCC 3.18353^{T}	KY742137	KY742293
	D. aliena	CBS 379.93 = PD 82/945	KP330416	GU237578
	D. americana	CBS 185.85 = PD 80/1191	KT389594	FJ427088
	D. anserina	CBS 253.80	KT389595	KT389795
	D. aquatica	CGMCC 3.18349^{T}	KY742140	KY742297
	D. arachidicola	CBS 333.75^{T} = ATCC 28333 = IMI 386092 = PREM 44889	KT389598	GU237554
	D. aurea	CBS 269.93^{T} = PD 78/1087	KT389599	GU237557
	D. bellidis	CBS 714.85 = PD 74/265	KP330417	GU237586
	D. boeremae	CBS 109942^{T} = PD 84/402	KT389600	FJ427097
	D. brunneospora	CBS 115.58 = DSM 62044	KT389625	KT389802
	D. calidophila	CBS 448.83^{T}		FJ427168
	D. chenopodii	CBS 128.93 = PD 79/140	KT389602	GU237591
	D. chloroguttulata	CGMCC 3.18351^{T}	KY742142	KY742299
	D. coffeae-arabicae	CBS 123380^{T} = PD 84/1013	KT389603	FJ427104
	D. corylicola	CREADC-F2281	MN958321	MN958331
	D. corylicola	CREADC-F2405	MN958324	MN958334
	D. corylicola	CREADC-F2406	MN958325	MN958335
	D. corylicola	CREADC-F2407	MN958326	MN958336
	D. corylicola	CREADC-F2408	MN958327	MN958337
	D. curtisii	CBS 251.92 = PD 86/1145		FJ427148
	D. dactylidis	CBS 124513^{T} = PD 73/1414		GU237599
	D. dimorpha	CBS 346.82^{T}		GU237606
	D. ellipsoidea	CGMCC 3.18350^{T}	KY742145	KY742302
	D. eucalyptica	CBS 377.91 = PD 79/210	KT389605	GU237562
	D. exigua	CBS 183.55^{T}	EU874850	GU237525

Supplementary Table 4 continued. *Didymella* sampling informed by Chen et al. (2017) and Scarpari et al. (2020).

	Name	Voucher	RPB2	TUB2
***Didymella* (Figure 3A)**	*D. gardeniae*	CBS 626.68^{T} = IMI 108771	KT389606	FJ427114
	D. glomerata	CBS 133.72		FJ427115
	D. glomerata	CBS 528.66^{ET} = PD 63/590	GU371781	FJ427124
	D. glomerata	ATCC MYA-2373	MZ073895	MZ073910
	D. glomerata	CBS 126930	MN983465	MN983856
	D. glomerata	UTHSC:DI16-205	LT593043	LT592974
	D. heteroderae	CBS 109.92^{T} = PD 73/1405	KT389601	FJ427098
	D. ilicicola	CGMCC 3.18355^{T}	KY742150	KY742307
	D. ilicicola	LC 8127	KY742151	KY742308
	D. infuscatispora	CGMCC 3.18356^{T}	KY742152	KY742309
	D. keratinophila	CBS 143032 = UTHSC:DI16–200 = FMR 13690	LT593039	LT592970
	D. lethalis	CBS 103.25	KT389607	GU237564
	D. longicolla	CBS 124514^{T} = PD 80/1189		GU237622
	D. longicolla	CBS 503.71	MN983480	MN983866
	D. longicolla	CBS 347.82	MT018160	GU237621
	D. macrophylla	CGMCC 3.18357^{T}	KY742154	KY742312
	D. macrostoma	CBS 482.95	KT389609	GU237626
	D. maydis	CBS 588.69^{T}	GU371782	FJ427190
	D. microchlamydospora	CBS 105.95^{T}	KP330424	FJ427138
	D. molleriana	CBS 229.79 = LEV 7660	KP330418	GU237605
	D. musae	CBS 463.69	LT623248	FJ427136
	D. negriana	CBS 358.71	KT389610	GU237635
	D. nigricans	CBS 444.81^{T} = PDDCC 6546		GU237558
	D. ocimicola	CGMCC 3.18358^{T}		KY742320
	D. pedeiae	CBS 124517^{T} = PD 92/612A	KT389612	GU237642
	D. pinodella	CBS 531.66	KT389613	FJ427162
	D. pinodes	CBS 525.77^{T}	KT389614	GU237572

Supplementary Table 4 continued. *Didymella* sampling informed by Chen et al. (2017) and Scarpari et al. (2020).

	Name	Voucher	RPB2	TUB2
***Didymella* (Figure 3A)**	*D. pomorum*	CBS 285.76 = ATCC 26241 = IMI 176742 = VKM F-1843	KT389615	FJ427163
	D. pomorum	CBS 388.80	KT389617	FJ427165
	D. pomorum	CBS 539.66 = ATCC 16791 = IMI 122266 = PD 64/914	KT389618	FJ427166
	D. pomorum	CBS 354.52	KT389616	KT389799
	D. protuberans	CBS 381.96^{T} = PD 71/706	KT389620	GU237574
	D. pteridis	CBS 379.96^{T}	KT389624	KT389801
	D. rhei	CBS 109177 = LEV 15165 = PD 2000/9941	KP330428	GU237653
	D. rosea	BRIP 50788		KT286945
	D. rumicicola	CBS 683.79^{T} = LEV 15094	KT389622	KT389800
	D. sancta	CBS 281.83^{T}	KT389623	FJ427170
	D. segeticola	CGMCC 3.17489^{T}	KP330414	KP330399
	D. senecionicola	CBS 160.78 = LEV 11451		GU237657
	D. sinensis	LC 8142	KY742166	KY742329
	D. subglomerata	CBS 110.92 = PD 76/1010	KT389626	FJ427186
	D. subherbarum	CBS 250.92^{T} = DAOM 171914 = PD 92/371		GU237659
	D. subherbarum	CBS 249.92 = PD 78/1088		GU237658
	D. suiyangensis	CGMCC 3.18352^{T}	KY742168	KY742331
	D. tanaceti	BRIP 50785		KT286974
	D. viburnicola	CBS 523.73 = PD 69/800	KP330430	GU237667
	Macroventuria anomochaeta	CBS 525.71	GU456346	GU237544
	Macroventuria wentii	CBS 526.71	KT389642	GU237546
	Paraboeremia adianticola	CBS 187.83 = PD 82/128	KP330401	GU237576
	Paraboeremia putaminum	CBS 130.69 = CECT 20054 = IMI 331916	LT623254	GU237652
	Paraboeremia selaginellae	CBS 122.93 = PD 77/1049	LT623255	GU237656

Supplementary Table 4 continued. *Didymosphaeriaceae* sampling informed by Karácsony et al. (2021) and Wanasinghe and Mortimer (2022).

	Name	Voucher	LSU	RPB2	TEF1	TUB2
***Didymosphaeriaceae* (Figure 3D)**	*Alloconiothyrium camelliae*	NTUCC 17-032-1^{T}	MT071270		MT232967	MT308624
	Austropleospora keteleeriae	MFLUCC 18-1551^{T}	NG_070075	MK434909	MK360045	
	Austropleospora ochracea	KUMCC 20-0020^{T}	MT799860		MT872714	
	Austropleospora osteospermi	MFLUCC 17-2429^{T}	MK347974	MK434884	MK360044	
	Bambusistroma didymosporum	MFLU 15-0057^{T}	KP761730	KP761720	KP761727	
	Bimuria novae-zelandiae	CBS 107.79^{T}	AY016356	DQ470917	DQ471087	
	Bimuria omanensis	SQUCC 15280^{T}	NG_071257		MT279046	
	Chromolaenicola lampangensis	MFLUCC 17-1462^{T}	MN325004	MN335654	MN335649	
	Chromolaenicola nanensis	MFLUCC 17-1477	MN325002	MN335653	MN335647	
	Chromolaenicola thailandensis	MFLUCC 17-1475^{T}	MN325007	MN335656	MN335652	
	Cylindroaseptospora leucaenae	MFLUCC 17-2424^{T}	NG_066310		MK360047	
	Cylindroaseptospora siamensis	MFLUCC 17-2527	NG_066311		MK360048	
	Deniquelata barringtoniae	MFLUCC 16-0271	MH260291	MH412753	MH412766	
	Deniquelata hypolithi	CBS 146988	MZ064486	MZ078201	MZ078250	
	Deniquelata vittalii	NFCCI4249^{T}	MF182395	MF168942	MF182398	
	Didymocrea sadasivanii	CBS 438.65^{T}	DQ384103			
	Didymosphaeria rubi ulmifolii	CBS 100299	JX496124			JX496350
	Didymosphaeria rubi ulmifolii	MFLUCC 14-0023^{T}	KJ436586			KJ939277
	Didymosphaeria variabile	18EPLE013			MT881834	MT881920
	Didymosphaeria variabile	CBS 638.93	JX496215			JX496441
	Didymosphaeria variabile	18EPLE021			MT881841	MT881928
	Kalmusia cordylines	ZHKU 21-0003	OL818333			
	Kalmusia ebuli	CBS 123120^{T}	JN644073			
	Kalmusia erioi	MFLU 18-0832	MN473052		MN481599	MN481603
	Kalmusia italica	MFLUCC 14-0566	KP325441			
	Kalmusia longispora	CBS 582.83^{T}	MH873371			JX496436
	Kalmusia sarothamni	CBS 116474	KF796673			
	Kalmusia sarothamni	CBS 113833	KF796671			

Supplementary Table 4 continued. *Didymosphaeriaceae* sampling informed by Karácsony et al. (2021) and Wanasinghe and Mortimer (2022).

***Didymosphaeriaceae* (Figure 3D)**

Name	Voucher	LSU	RPB2	TEF1	TUB2
Kalmusia sp.	K			MW692012	MW692021
Kalmusia sp.	UTHSC DI16-256	LN907399	LT797014	LT797094	LT796934
Kalmusia variisporum	CBS 121517^{T}	JX496143			JX496369
Kalmusibambusa triseptata	MFLUCC 13-0232^{T}	KY682695			
Karstenula rhodostoma	CBS 690.94	GU301821	GU371788	GU349067	
Karstenula rhodostoma	CBS 691.94	AB807531		AB808506	
Laburnicola muriformis	MFLUCC 16-0290^{T}	KU743198		KU743213	KU743214
Laburnicola rhizohalophila	CGMCC 8756	KJ125523	KJ125524	KJ125525	
Letendraea cordylinicola	MFLUCC 11-0148^{T}	NG_059530			
Letendraea helminthicola	CBS 884.85	AY016362	MK404164	MK404174	
Letendraea padouk	CBS 485.70	AY849951			
Montagnula bellevaliae	MFLUCC 14-0924^{T}	KT443902		KX949743	
Montagnula chromolaenicola	MFLUCC 17-1469	NG_070948	MT235809	MT235773	
Montagnula cirsii	MFLUCC 13-0680^{T}	KX274249		KX284707	
Montagnula krabiensis	MFLUCC 16-0250^{T}	MH260303		MH412776	
Montagnula thailandica	MFLUCC 17-1508	NG_070949	MT235810	MT235774	
Neokalmusia arundinis	MFLU 17-0754	MT649878		MT663766	
Neokalmusia brevispora	KT 2313	AB524601	AB539100	AB539113	
Neokalmusia didymospora	MFLUCC 11-0613	KP091434			
Neokalmusia jonahhulmei	KUMCC 21-0818	ON007039	ON009137	ON009133	
Neokalmusia kunmingensis	KUMCC 18-0120	MK079889		MK070172	
Neokalmusia scabrispora	KT 1023	AB524593	AB539093	AB539106	
Neokalmusia thailandica	MFLUCC 16-0405	NG_059792	KY706148	KY706145	
Neptunomyces aureus	CMG12			MK948000	MK934132
Paracamarosporium hawaiiense	CBS 120025^{T}	JX496140			JX496366
Paraconiothyrium cyclothyrioides	CBS 972.95^{T}	JX496232			JX496458
Paraconiothyrium estuarinum	CBS 109850^{T}	JX496129			JX496355

Supplementary Table 4 continued. *Didymosphaeriaceae* sampling informed by Karácsony et al. (2021) and Wanasinghe and Mortimer (2022).

	Name	Voucher	LSU	RPB2	TEF1	TUB2
***Didymosphaeriaceae* (Figure 3D)**	*Paramassariosphaeria anthostomoides*	CBS 615.86	GU205223			
	Paramassariosphaeria clematidicola	MFLU 16-0172^{T}	KU743207			
	Paraphaeosphaeria rosae	MFLUCC 17-2547^{T}	MG829044		MG829222	
	Phaeodothis winteri	CBS 182.58	GU301857			
	Pseudocamarosporium eucalypti	CBS 146084^{T} = CPC 37995	MN567657		MN556833	
	Pseudocamarosporium pteleae	MFLUCC 17-0724^{T}	MG829061		MG829233	
	Pseudodidymocyrtis lobariellae	KRAM Flakus 25130^{T}	NG_068933			
	Pseudopithomyces entadae	MFLUCC 17-0917^{T}	NG_066305	MK434899	MK360083	
	Pseudopithomyces kunmingnensis	MFLUCC 17-0314^{T}	MF173605			
	Pseudopithomyces rosae	MFLUCC 15-0035^{T}	MG829064			
	Spegazzinia radermacherae	MFLUCC 17-2285^{T}	MK347957	MK434893	MK360088	
	Spegazzinia tessarthra	SH 287	AB807584		AB808560	
	Tremateia arundicola	MFLU 16-1275^{T}	KX274248		KX284706	
	Tremateia chromolaenae	MFLUCC 17-1425	NG_068710	MT235816	MT235778	
	Tremateia guiyangensis	GZAAS01^{T}	KX274247		KX284705	
	Tremateia murispora	GZCC 18-2787^{T}	MK972751		MK986482	
	Tremateia thailandensis	MFLUCC 17-1430	NG_068711	MT235819	MT235781	
	Verrucoconiothyrium nitidae	CBS 119209	EU552112			
	Xenocamarosporium acaciae	CPC 24755^{T}	NG_058163			
	Xenocamarosporium acaciae	MFLUCC 17-2432	MK347983		MK360093	

Supplementary Table 4 continued. *Gnomoniopsis* sampling informed by Jiang et al. (2021).

	Name	Voucher	TEF1	TUB2
***Gnomoniopsis* (Figure 3F)**	*Gnomoniopsis alderdunensis*	CBS 125680^{T}	GU320801	GU320787
	G. castanopsidis	CFCC 54437^{T}	MZ936385	
	G. chamaemori	CBS 804.79	GU320809	GU320777
	G. chinensis	CFCC 52286^{T}	MH545370	MH545366
	G. chinensis	CFCC 52287	MH545371	MH545367
	G. chinensis	CFCC 52288	MH545372	MH545368
	G. chinensis	CFCC 52289	MH545373	MH545369
	G. clavulata	CBS 121255	GU320807	EU219211
	G. clavulata	AR 4124	EU221977	EU219167
	G. clavulata	AR 4183	EU221965	EU219190
	G. clavulata	AR 4317 = BPI 877443	EU221938	EU219214
	G. comari	CBS 806.79	GU320810	EU219156
	G. daii	CFCC 54043^{T}	MN605519	MN605517
	G. fagacearum	CFCC 54316^{T}	MZ936392	MZ936408
	G. fragariae	CBS 121226	GU320792	EU219144
	G. guangdongensis	CFCC 54443^{T}	MZ936394	MZ936410
	G. hainanensis	CFCC 54376^{T}	MZ936397	MZ936413
	G. idaeicola	CBS 125672	GU320797	GU320781
	G. macounii	CBS 121468	GU320804	EU219126
	G. occulta	CBS 125677	GU320812	GU320785
	G. paraclavulata	CBS 123202	GU320815	GU320775
	G. paraclavulata	66G	MZ078875	MZ078820
	G. paraclavulata	477E	MZ078874	MZ078819
	G. paraclavulata	396E	MZ078873	MZ078818
	G. racemula	CBS 121469^{T}	GU320803	EU219125
	G. rossmaniae	CFCC 54307^{T}	MZ936399	MZ936415
	G. sanguisorbae	CBS 858.79	GU320805	GU320790
	G. silvicola	CFCC 54418^{T}	MZ936402	MZ936418

Supplementary Table 4 continued. *Gnomoniopsis* sampling informed by Jiang et al. (2021).

Name	Voucher	TEF1	TUB2
G. smithogilvyi	CBS 130190^{T}	KR072534	JQ910639
G. smithogilvyi	CBS 130189	KR072535	JQ910641
G. smithogilvyi	CBS 130188	KR072536	JQ910640
G. smithogilvyi	MUT 401	KR072537	KR072532
G. smithogilvyi	MUT 411	KR072538	KR072533
G. tormentillae	CBS 904.79	GU320795	EU219165
G. xunwuensis	CFCC 53115^{T}	MK578141	MK578067
Melanconis alni	AR 3500	EU221896	EU219102
M. marginalis	AR 3442	EU221991	EU219103

Supplementary Table 4 continued. *Neocosmospora* sampling informed by Crous, Lombard, et al. (2021).

	Name	Voucher	acl1	cmdA	RPB1	RPB2	TEF1
***Neocosmospora* (Figure 3J)**	*Geejayessia atrofusca*	CBS 125482 = DAOM 238117			MW834196	HQ897775	MW834282
	G. celtidicola	CBS 125502^{T}	HM626625		MW834197	MW834013	HM626638
	G. cicatricum	CBS 125550			MW834198	HQ897697	HM626642
	Neocosmospora acutispora	CBS 145461^{T} = NRRL 22574 = BBA 62213	MW834050	MW834122	MW834210	LR583814	LR583593
	Neoco. addoensis	CBS 146510^{T} = CPC 37128	MW218005	MW218052	MW218098	MW446575	MW248741
	Neoco. ambrosia	CBS 571.94^{ET} = NRRL 22346 = BBA 65390 = MAFF 246287			MW834211	EU329503	FJ240350
	Neoco. ampla	CBS 202.32^{T} = BBA 4170	MW834051	MW834123	MW834212	LR583815	LR583594
	Neoco. bataticola	CBS 144398^{T} = NRRL 22402 = BBA 64954 = FRC S-0567	MW218007	MW218054	MW218100	FJ240381	AF178344
	Neoco. borneensis	CBS 145462^{ET} = NRRL 22579 = BBA 65095 = GJS 85-197	MW834052	MW834124	MW834213	EU329515	AF178352
	Neoco. bostrycoides	CBS 144.25 NT	MW218008	MW218055	MW218101	LR583818	LR583597
	Neoco. brevicona	CBS 204.31^{ET} = NRRL 22659 = BBA 2123	MW218010	MW218057	MW218103	LR583821	LR583600
	Neoco. brevis	CBS 130326 = NRRL 28009 = CDC B-5543	MW834053	MW834125	MW834214	EF470136	DQ246869
	Neoco. catenata	CBS 143229^{T} = NRRL 54993 = U THSC 09-1009	MW218012	MW218059	MW218105	KC808355	KC808214
	Neoco. citricola	CBS 146513^{T} = CPC 37131	MW218015	MW218062	MW218108	MW446581	MW248747
	Neoco. crassa	CBS 144386^{T} = MUCL 11420	MW218016	MW218063	MW218109	LR583823	LR583604
	Neoco. cryptoseptata	CBS 145463^{T} = NRRL 22412 = BBA 65024	MW834054	MW834126	MW834215	EU329510	AF178351
	Neoco. cucurbitae	CBS 410.62 = NRRL 22658 = CECT 2864	MW834055	MW834127	MW834216	LR583824	DQ247640
	Neoco. cucurbitae	CBS 616.66^{T} = NRRL 22399 = BBA 64411	MW834056	MW834128	MW834217	LR583825	DQ247592
	Neoco. cyanescens	CBS 518.82^{T}	MW218017	MW218064	MW218110	LR583826	LR583605
	Neoco. diminuta	CBS 144390^{T} = MUCL 18798	MW834057	MW834129	MW834218	LR583828	LR583607
	Neoco. elegans	CBS 144396^{ET} = NRRL 22277 = MAFF 238541 = ATCC 42366	MW218020	MW218067	MW218113	FJ240380	AF178336
	Neoco. epipeda	CBS 146523^{T} = CPC 38310	MW834058	MW834130	MW834219	MW834022	MW834285
	Neoco. euwallaceae	CBS 135854^{T} = NRRL 54722			JQ038021	JQ038028	JQ038007
	Neoco. falciformis	CBS 475.67^{T} = IMI 268681	MW218021	MW218068	MW218114	LT960558	LT906669

Supplementary Table 4 continued. *Neocosmospora* sampling informed by Crous, Lombard, et al. (2021).

	Name	Voucher	acl1	cmdA	RPB1	RPB2	TEF1
***Neocosmospora* (Figure 3J)**	*Neoco. ferruginea*	CBS 109028^{T} = NRRL 32437	MW834060	MW834132	MW834221	EU329581	DQ246979
	Neoco. floridana	NRRL 62628^{T} = MAFF 246849			KC691593	KC691624	KC691535
	Neoco. gamsii	CBS 143207^{T} = NRRL 32323 = UTHSC 99-205	MW834062	MW834134	MW834223	EU329622	DQ247103
	Neoco. gamtoosensis	CBS 146502^{T} = VG16 = CPC 37120	MW218023	MW218070	MW218116	MW446611	MW248762
	Neoco. haematococca	CBS 119600^{ET} = FRC S-1832	MW834064	MW834136		LT960561	DQ247510
	Neoco. hypothenemi	CBS 145464^{T} = NRRL 52782 = ARSEF 5878	MW218024		MW218117	JF741176	JF740850
	Neoco. illudens	CBS 147303 = NRRL 22090 = BBA 67606 = GJS 82-98	MW834065	MW834137	JX171488	JX171601	AF178326
	Neoco. ipomoeae	CBS 353.87 = NRRL 22657	MW218026	MW218072	MW218119	LR583831	DQ247639
	Neoco. keleraja	CBS 125720 PT = FRC S-1837 = GJS 02-114	MW834066	MW834138	MW834225	LR583834	LR583612
	Neoco. keratoplastica	CBS 490.63^{T}	MW218028	MW218074	MW218121	LT960562	LT906670
	Neoco. kuroshio	CBS 142642^{T}	MW834068	MW834140	MW834227	LR583837	KX262216
	Neoco. kurunegalensis	CBS 119599^{T} = GJS 02-94	MW834069	MW834141	MW834228	LR583838	DQ247511
	Neoco. lerouxii	CBS 146514^{T} = CPC 37132	MW218030	MW218076	MW218123	MW446617	MW248768
	Neoco. lichenicola	CBS 623.92^{ET}	MW834071	MW834143		LR583845	LR583620
	Neoco. liriodendri	CBS 117481^{T} = NRRL 22389 = BBA 67587 = GJS 91-148	MW218031	MW218077	MW218124	EU329506	AF178340
	Neoco. longissima	CBS 126407^{T} = GJS 85-72	MW834072	MW834144	MW834230	LR583846	LR583621
	Neoco. macrospora	CBS 142424^{T} = CPC 28191	MW218032	MW218078	MW218125	LT746331	LT746218
	Neoco. mahasenii	CBS 119594^{T}	MW834073	MW834145	MW834231	LT960563	DQ247513
	Neoco. martii	CBS 115659^{ET} = FRC S-0679 = MRC 2198	MW834074	MW834146	MW834232	JX435256	JX435156
	Neoco. merkxiana	CBS 146525^{T}	MW834075	MW834147	MW834233	MW834025	MW834288
	Neoco. metavorans	CBS 135789^{T}	MW218034	MW218080	MW218127	LR583849	LR583627
	Neoco. mori	CBS 145467^{T} = NRRL 22230 = MAFF 238539	MW834077	MW834149	MW834235	EU329499	AF178358
	Neoco. neerlandica	CBS 232.34^{T}	MW834079	MW834151	MW834237	MW847903	MW847906
	Neoco. nelsonii	CBS 309.75^{T}	MW834080	MW834152	MW834238	MW847904	MW847907
	Neoco. nirenbergiana	CBS 145469^{T} = NRRL 22387 = BBA 65023 = GJS 87-127	MW834081	MW834153		EU329505	AF178339

Supplementary Table 4 continued. *Neocosmospora* sampling informed by Crous, Lombard, et al. (2021).

	Name	Voucher	acl1	cmdA	RPB1	RPB2	TEF1
***Neocosmospora* (Figure 3J)**	*Neoco. noneumartii*	CBS 115658^{T} = FRC S-0661	MW218036	MW218082	MW218129	MW446618	LR583630
	Neoco. obliquiseptata	NRRL 62611 = MAFF 246845			KC691606	KC691637	KC691548
	Neoco. oblonga	CBS 130325^{T} = NRRL 28008 = CDC B-4701	MW834082	MW834154	MW834239	LR583853	LR583631
	Neoco. oligoseptata	CBS 143241^{T} = NRRL 62579 = FRC S-2581 = MAFF 246283	MW834083	MW834155	KC691596	LR583854	KC691538
	Neoco. paraeumartii	CBS 487.76^{T} = NRRL 13997 = BBA 62215	MW834084	MW834156	MW834240	LR583855	DQ247549
	Neoco. parceramosa	CBS 115695^{T}	MW218037	MW218083		JX435249	JX435149
	Neoco. perseae	CBS 144142^{T} = CPC 26829	MW218038	MW218084	MW218130	LT991909	LT991902
	Neoco. petroliphila	CBS 203.32 = NRRL 13952	MW218039	MW218085	MW218131	LR583857	DQ246835
	Neoco. phaseoli	CBS 265.50	MW834085	MW834157		KJ511278	FJ919464
	Neoco. piperis	CBS 145470^{T} = NRRL 22570 = GJS 89-14 = CML 1888	MW834086	MW834158	MW834241	EU329513	AF178360
	Neoco. piperis	CML 3171				KT943484	KT943486
	Neoco. piperis	CML 3178				KT943485	KT943487
	Neoco. pisi	CBS 123669^{ET} = NRRL 45880 = ATCC MYA-4622	MW834087	MW834159	MW834242	LR583862	LR583636
	Neoco. plagianthi	NRRL 22632 = GJS 83-146			JX171501	JX171614	AF178354
	Neoco. protoensiformis	CBS 145471^{T} = NRRL 22178 = GJS 90-168	MW834089	MW834161	MW834244	EU329498	AF178334
	Neoco. pseudensiformis	CBS 130.78 = NRRL 22575 = NRRL 22653	MW834090	MW834162	MW834245	LR583868	DQ247635
	Neoco. pseudopisi	CBS 266.50	MW834091	MW834163	MW834246	MW834027	MW834290
	Neoco. pseudoradicicola	CBS 145472^{T} = NRRL 25137 = ARSEF 2313	MW218041	MW218087	MW218133	JF741084	JF740757
	Neoco. quercicola	CBS 141.90^{T} = NRRL 22652	MW834092	MW834164	MW834247	LR583869	DQ247634
	Neoco. rectiphora	CBS 125727^{T} = GJS 02-89 = FRC S-1831	MW834094	MW834166	MW834249	LR583871	DQ247509
	Neoco. regularis	CBS 230.34^{T}	MW834096	MW834168		MW834029	LR583643
	Neoco. rekana	CMW 52862^{T}				MN249137	MN249151
	Neoco. robusta	CBS 145473^{T} = NRRL 22395 = BBA 65682		MW834169	MW834251	EU329507	AF178341
	Neoco. samuelsii	CBS 114067^{T} = GJS 89-70	MW834097	MW834170	MW834252	LR583874	LR583644
	Neoco. silvicola	CBS 123846^{T} = GJS 04-147	MW834099	MW834172	MW834254	LR583876	LR583646

Supplementary Table 4 continued. *Neocosmospora* sampling informed by Crous, Lombard, et al. (2021).

Name	Voucher	acl1	cmdA	RPB1	RPB2	TEF1
Neoco. solani	CBS 140079^{ET} = NRRL 66304 = GJS 09-1466 = FRC S-2364	MW218042	MW218088	MW218134	KT313623	KT313611
Neoco. spathulata	CBS 145474^{T} = NRRL 28541 = UTHSC 98-1305	MW218045	MW218091	MW218137	EU329542	DQ246882
Neoco. stercicola	CBS 142481^{T} = DSM 106211	MW834100	MW834173	MW834255	LR583887	LR583658
Neoco. suttoniana	CBS 143214^{T} = NRRL 32858	MW218046	MW218092	MW218138	EU329630	DQ247163
Neoco. tonkinensis	CBS 115.40^{T}	MW218048	MW218094	MW218140	LT960564	LT906672
Neoco. tuaranensis	NRRL 22231^{T} = ATCC 16563 = MAFF 246842			KC691600	KC691631	KC691542
Neoco. vasinfecta	CBS 533.65 = IMI 302625	MW834103	MW834176	MW834258	LR583899	LR583671

Supplementary Table 4 continued. *Neocucurbitaria* sampling informed by Wanasinghe, Phookamsak, et al. (2017), Jaklitsch et al. (2018), Valenzuela-Lopez et al. (2018), and Crous, Schumacher, et al. (2019).

	Name	Voucher	RPB2	TEF1	TUB2
***Neocucurbitaria* (Figure 3E)**	*Cucurbitaria berberidis*	CBS 142401 = C241	MF795798	MF795845	MF795886
	Cucurbitaria oromediterranea	CBS 142399 = C229^{T}	MF795803	MF795849	MF795890
	Neocucurbitaria acanthocladae	CBS 142398 = C225^{T}	MF795808	MF795854	MF795894
	Neocu. acerina	CBS 142403 = C255	MF795810	MF795856	MF795896
	Neocu. aetnensis	CBS 142404 = C261^{T}	MF795811	MF795857	MF795897
	Neocu. aquatica	CBS 297.74	LT623278		LT623238
	Neocu. cava	CBS 115979	LT623273		LT623234
	Neocu. cava	CBS 257.68^{T}	LT717681		KT389844
	Neocu. cava	CBS 143400	MH108005		MH108046
	Neocu. cava	MF-Vm17-040			MZ054692
	Neocu. cinereae	CBS 142406 = KU9^{T}	MF795813	MF795859	MF795899
	Neocu. cisticola	CBS 142402 = C244^{T}	MF795814	MF795860	MF795900
	Neocu. hakeae	CBS 142109	KY173593		KY173613
	Neocu. irregularis	CBS 142791	LT593054		LT592985
	Neocu. juglandicola	CBS 142390 = BW6^{T}	MF795815	MF795861	MF795901
	Neocu. keratinophila	CBS 121759^{T}	LT623275		LT623236
	Neocu. populi	CBS 142393 = C28^{T}	MF795816	MF795862	MF795902
	Neocu. quercina	CBS 115095^{T}	LT623277		LT623237
	Neocu. rhamni	CBS 142391 = C1^{T}	MF795817	MF795863	
	Neocu. rhamnicola	CBS 142396 = C185^{T}	MF795822	MF795868	MF795906
	Neocu. rhamnioides	CBS 142395 = C118^{T}	MF795824	MF795870	MF795908
	Neocu. ribicola	CBS 142394 = C55^{T}	MF795827	MF795873	MF795911
	Neocu. unguis-hominis	CBS 111112	LT623279		LT623239
	Neocu. vachelliae	CBS 142397 = C192^{T}	MF795829	MF795875	MF795913
	Neopyrenochaeta acicola	CBS 812.95^{T}	LT623271		LT623232
	Parafenestella pseudoplatani	CBS 142392 = C26^{T}	MF795830	MF795876	MF795914
	Protofenestella ulmi	CBS 143000 = FP5^{T}	MF795833	MF795879	MF795915

Supplementary Table 4 continued. *Neodidymelliopsis* sampling informed by Chen et al. (2017), Hyde et al. (2019), and Hou et al. (2020).

	Name	Voucher	RPB2	TUB2
***Neodidymelliopsis* (Figure 3C)**	*Calophoma clematidina*	CBS 108.79	KT389588	FJ427100
	Calophoma complanata	CBS 100311	KT389590	GU237594
	Calophoma glaucii	CBS 112.96	MT018230	GU237610
	Neoascochyta cylindrispora	UTHSC DI16-352	LT593101	LT593031
	Neoascochyta desmazieri	CBS 346.86	MT018304	MT005730
	Neoascochyta europaea	CBS 504.71	MT018314	MT005738
	Neodidymelliopsis achlydis	CBS 256.77^{T}		KT389829
	Neod. cannabis	CBS 121.75^{T}		GU237535
	Neod. cannabis	CBS 234.37	KP330403	GU237523
	Neod. cannabis	CBS 591.67		KT389826
	Neod. cannabis	CBS 629.76		KT389827
	Neod. farokhinejadii	CBS 142853	KY464922	KY449023
	Neod. longicolla	CBS 382.96^{T}	MT018298	KT389830
	Neod. sp.	CBS 141235		KX033382
	Neod. longicolla	CBS 265.74	MT018296	MT005725
	Neod. longicolla	CBS 266.74	MT018297	MT005726
	Neod. moricola	MFLUCC 17-1063	KY684943	KY684937
	Neod. moricola	MFLUCC 17-1064^{T}	KY684944	KY684938
	Neod. negundinis	MFLUCC 18-0083^{T}	MG564166	MG564164
	Neod. polemonii	CBS 109181^{T} = PD 83/757	KP330427	GU237648
	Neod. polemonii	CBS 375.67	MT018291	KT389828
	Neod. ranunculi	CBS 739.88	MT018295	MT005724
	Neod. ranunculi	CBS 286.72	MT018294	MT005723
	Neod. sambuci	MFLUCC 18-1565		MK049556
	Neod. tiliae	CBS 139719	MT018286	MT005720
	Neod. tiliae	CBS 519.95^{T}	MT018287	MT005721
	Neod. xanthina	CBS 383.68^{T}	KP330431	GU237688
	Neod. xanthina	CBS 168.70	MT018290	KT389831

Supplementary Table 4 continued. *Neodidymelliopsis* sampling informed by Chen et al. (2017), Hyde et al. (2019), and Hou et al. (2020).

Name	Voucher	RPB2	TUB2
Xenodidymella asphodeli	CBS 499.72	MT018282	KT389853
Xenodidymella catariae	CBS 102635	KP330404	GU237524
Xenodidymella humicola	CBS 220.85	KP330422	GU237617
Xenodidymella applanata	CBS 195.36	MT018280	KT389852

Supplementary Table 4 continued. *Neurospora* sampling informed by Nygren et al. (2011).

	Name	Voucher	Bml	LSU	mak-2	nik-1	PKC	TEF1
***Neurospora* (Figure 3I)**	*Neurospora africana*	FMR 7370	FR774319	FR774244		FR774462	FR774484	FR774369
	Neu. brevispora	FGSC 7795	FR774295	FR774245	FR774394	FR774438	FR774485	FR774345
	Neu. calospora	FGSC 958	FR774296	FR774246	FR774395	FR774439	FR774486	FR774346
	Neu. cerealis	FGSC 959	FR774297	FR774247	FR774396	FR774440	FR774487	FR774347
	Neu. crassa	FGSC 8858	FR774322	FR774250	FR774419	FR774464	FR774490	FR774371
	Neu. crassa	FGSC 8771	FR774321	FR774249	FR774418	FR774463	FR774489	FR774370
	Neu. crassa	FGSC 959	FR774320	FR774248	FR774392	FR774436	FR774488	FR774343
	Neu. dictyophora	FMR 7511	FR774298	FR774251	FR774397	FR774441	FR774491	FR774348
	Neu. discreta	FGSC 8780	FR774332	FR774252	FR774426	FR774474	FR774492	FR774381
	Neu. dodgei	FGSC 1692	FR774323	FR774253		FR774465	FR774493	FR774372
	Neu. endodonta	IMI 148369^T	FR774299	FR774254	FR774398	FR774442	FR774494	FR774349
	Neu. galapagosensis	FGSC 1739	FR774324	FR774255		FR774466	FR774495	FR774373
	Neu. hapsidophora	CBS 408.82^T	FR774300	FR774256	FR774399	FR774443	FR774496	FR774350
	Neu. hispaniola	FGSC 8817	FR774329	FR774257	FR774423	FR774471	FR774497	FR774378
	Neu. indica	FGSC 7793	FR774301	FR774258	FR774400	FR774444	FR774498	FR774351
	Neu. intermedia	FGSC 8844	FR774326	FR774260	FR774421	FR774468	FR774500	FR774375
	Neu. intermedia	FGSC 8901	FR774325	FR774259	FR774420	FR774467	FR774499	FR774374
	Neu. kobi	CBS 560.72^T	FR774302	FR774261	FR774401	FR774445	FR774501	FR774352
	Neu. lineolata	CBS 502.70	FR774327	FR774262		FR774469	FR774502	FR774376
	Neu. metzenbergii	FGSC 8847	FR774330	FR774263	FR774424	FR774472	FR774503	FR774379
	Neu. minuta	FMR 7512	FR774303	FR774264	FR774402	FR774446	FR774504	FR774353
	Neu. nigeriensis	FMR 5963	FR774304	FR774265	FR774403	FR774447	FR774505	FR774354
	Neu. novoguineensis	FMR 7269	FR774305	FR774266	FR774404	FR774448	FR774506	FR774355
	Neu. pannonica	FGSC 7221	FR774328	FR774267	FR774422	FR774470	FR774507	FR774377
	Neu. perkinsii	FGSC 8838	FR774331	FR774268	FR774425	FR774473	FR774508	FR774380
	Neu. pseudoreticulata	CBS 556.72	FR774306	FR774269	FR774405	FR774449	FR774509	FR774356
	Neu. reticulata	IMI 080035^T	FR774307	FR774270	FR774406	FR774450	FR774510	FR774357
	Neu. reticulospora	FGSC 6537	FR774308	FR774271	FR774407	FR774451	FR774511	FR774358

Supplementary Table 4 continued. *Neurospora* sampling informed by Nygren et al. (2011).

	Name	Voucher	Bml	LSU	mak-2	nik-1	PKC	TEF1
***Neurospora* (Figure 3I)**	*Neu. retispora*	FMR 7510	FR774309	FR774272	FR774408	FR774452	FR774512	FR774359
	Neu. retispora	FMR 7276		AJ579677				
	Neu. retispora	FMR 5513		AJ579544				
	Neu. retispora	CBS 868.68		MH878403				
	Neu. retispora	CBS 656.70		MH871676				
	Neu. retispora	CBS 275.50^{T}		MH868127				
	Neu. saitoi	CBS 435.74^{T}	FR774311.2	FR774273	FR774410	FR774454	FR774513	FR774361
	Neu. santi-florii	FGSC 8331	FR774310	FR774274	FR774409	FR774453	FR774514	FR774360
	Neu. sitophila	FGSC 8770	FR774333	FR774275	FR774427	FR774475	FR774515	FR774382
	Neu. sp.	FGSC 8243	FR774315	FR774279	FR774414	FR774458	FR774519	FR774365
	Neu. sp.	FGSC 8240	FR774314	FR774278	FR774413	FR774457	FR774518	FR774364
	Neu. sp.	FGSC 8238	FR774313	FR774277	FR774412	FR774456	FR774517	FR774363
	Neu. sp.	FGSC 6877	FR774312	FR774276	FR774411	FR774455	FR774516	FR774362
	Neu. stellata	IFO 30242^{T}	FR774316	FR774280	FR774415	FR774459	FR774520	FR774366
	Neu. sublineolata	IMI 22388^{T}	FR774334	FR774281	FR774428	FR774476	FR774521	FR774383
	Neu. terricola	CBS 298.63^{T}	FR774335.2	FR774282	FR774429	FR774477	FR774522	FR774384
	Neu. tetrasperma	FMR 5545	FR774336	FR774283	FR774430	FR774478	FR774523	FR774385
	Neu. tetraspora	FGSC 7033	FR774317	FR774284	FR774416	FR774460	FR774524	FR774367
	Neu. udagawae	CBS 309.91^{T}	FR774318	FR774285	FR774417	FR774461	FR774525	FR774368
	Neu. uniporata	FMR 7283	FR774337	FR774286	FR774431	FR774479	FR774526	FR774386
	Pseudoneurospora amorphoporcata	CBS 626.80^{T}	FR774294	FR774287	FR774393	FR774437	FR774527	FR774344
	Sordaria brevicollis	FGSC 1904	FR774338	FR774288	FR774432	FR774480	FR774528	FR774387
	S. fimicola	FGSC 2918	FR774339	FR774289			FR774529	FR774388
	S. macrospora	FGSC 4818	FR774340	FR774290	FR774433	FR774481	FR774530	FR774389
	S. sclerogenia	FGSC 2741	FR774341	FR774291	FR774434	FR774482	FR774531	FR774390
	S. tomento-alba	CBS 260.78	FR774342.2	FR774292	FR774435	FR774483	FR774532	FR774391

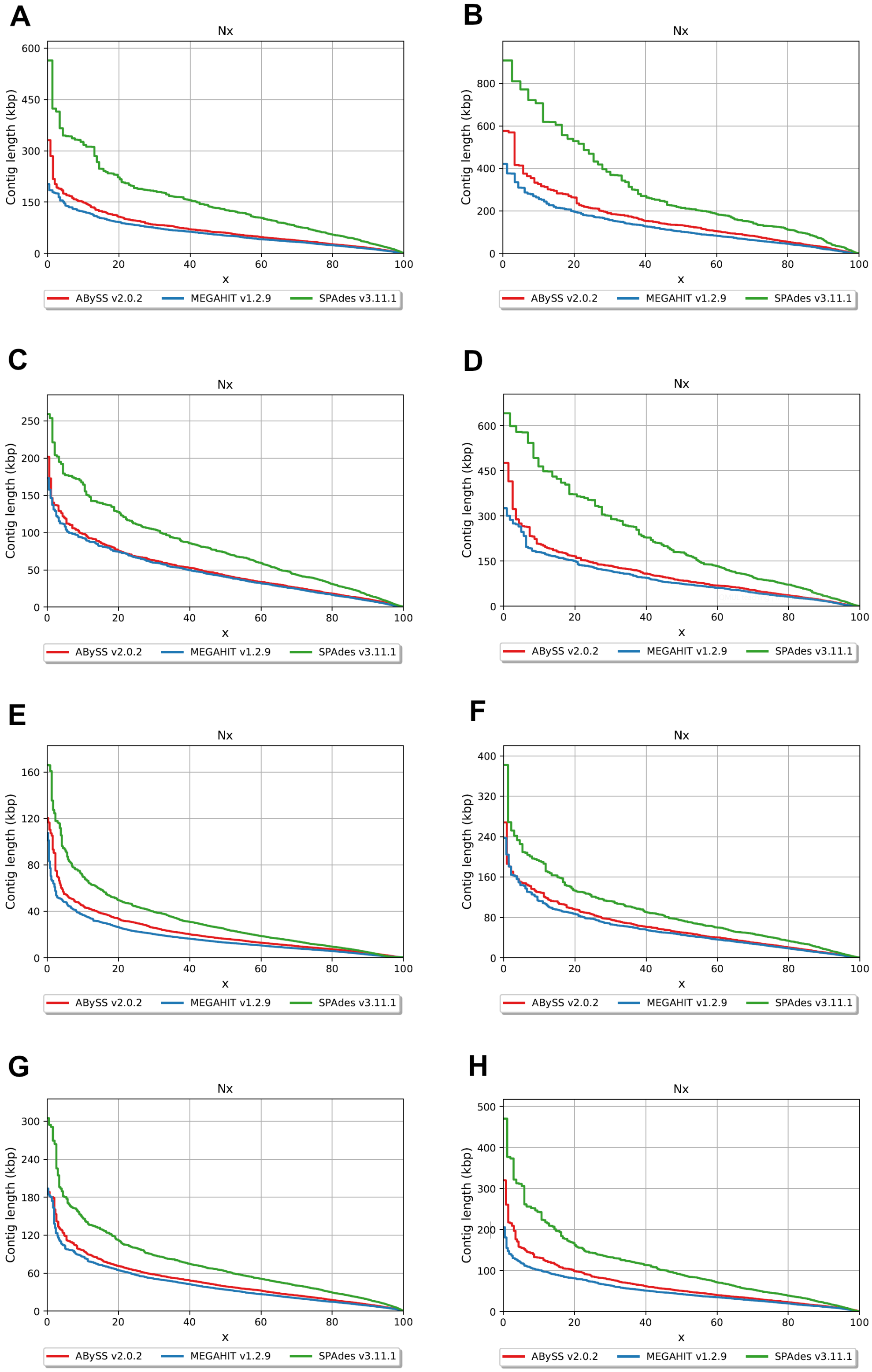

Supplementary Figure 1: Nx plots (the smallest contig length at which x% of the assembly is contained in contigs of at least that size) produced by QUAST for each of the strains sequenced in this chapter. Short-read assemblies: **(A)** IMI 355080 **(B)** IMI 355091 **(C)** IMI 359910 **(D)** IMI 360193 **(E)** IMI 360204 **(F)** IMI 364377 **(G)** IMI 366226 **(H)** IMI 366586. ▼

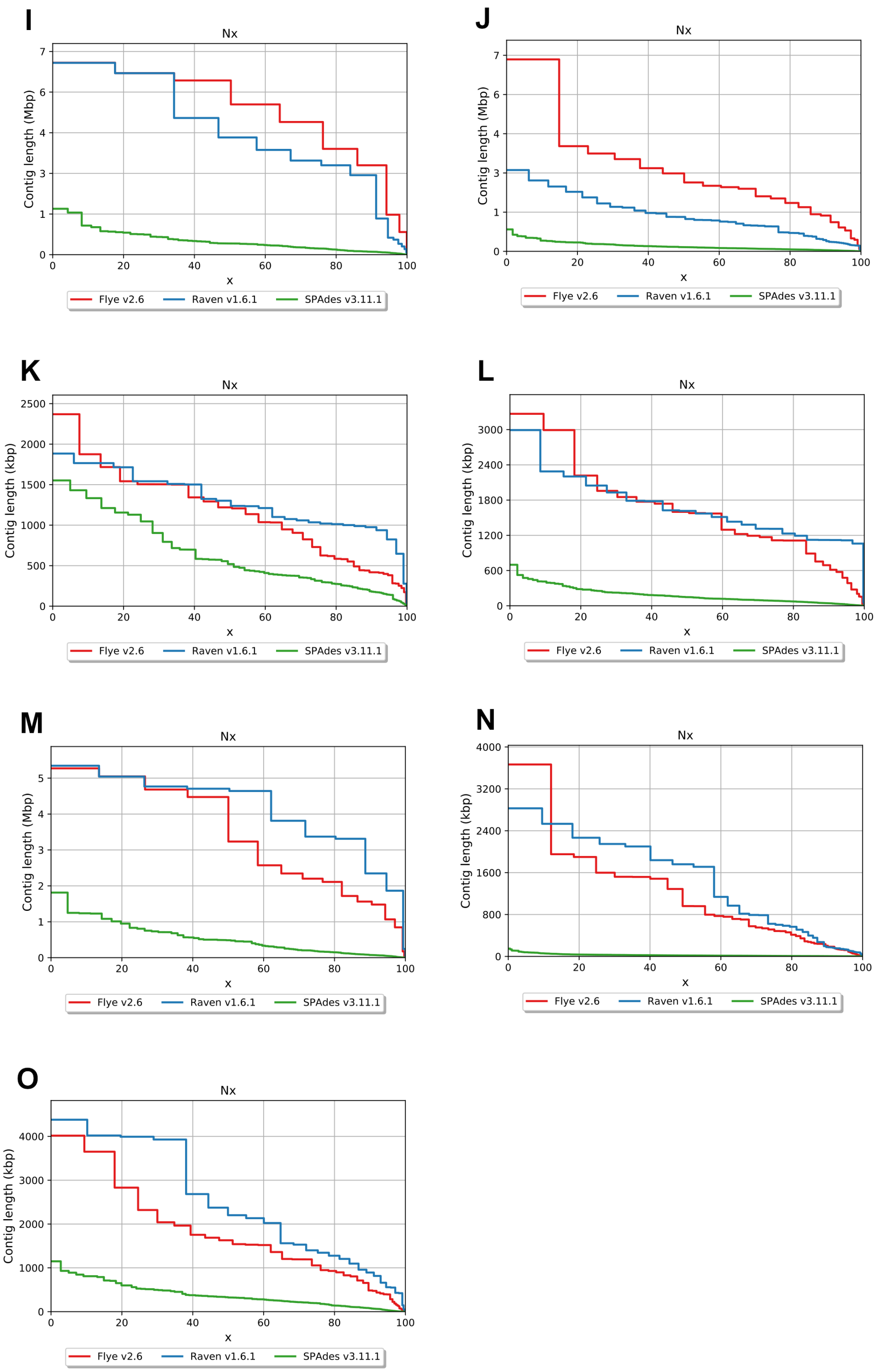

Supplementary Figure 1: continued. Hybrid assemblies: **(I)** IMI 355082 **(J)** IMI 355084 **(K)** IMI 355093 **(L)** IMI 356814 **(M)** IMI 356815 **(N)** IMI 366227 **(O)** IMI 367209.

A

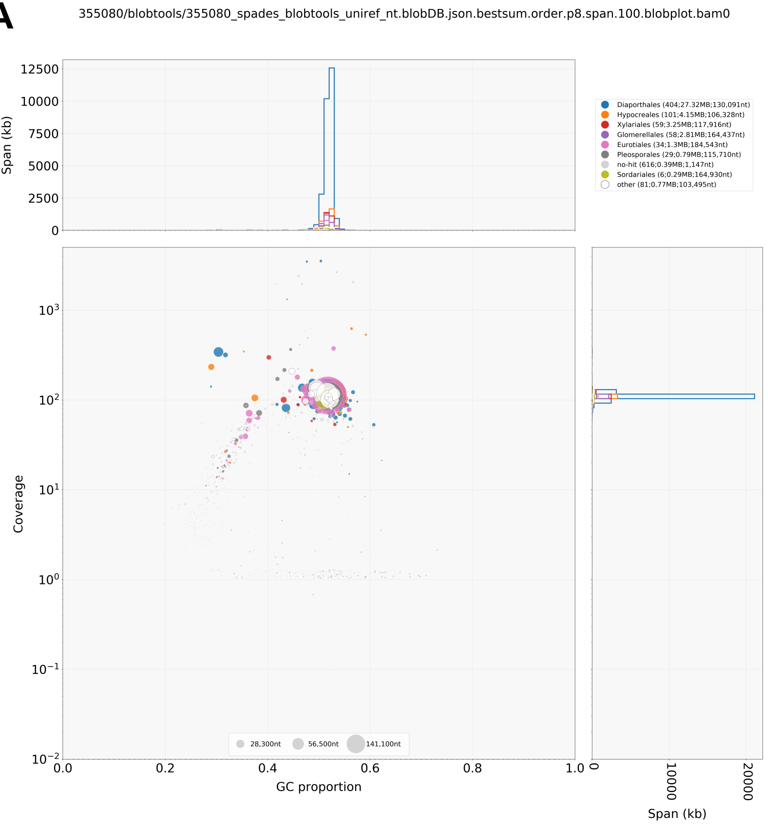

Supplementary Figure 2: BlobPlots for the 15 endophyte strains showing the taxonomic classification of reads based on coverage and GC content. Short-read assemblies: **(A)** IMI 355080 **(B)** IMI 355091 **(C)** IMI 359910 **(D)** IMI 360193 **(E)** IMI 360204 **(F)** IMI 364377 **(G)** IMI 366226 **(H)** IMI 366586. Hybrid assemblies: **(I)** IMI 355082 **(J)** IMI 355084 **(K)** IMI 355093 **(L)** IMI 356814 **(M)** IMI 356815 **(N)** IMI 366227 **(O)** IMI 367209. ▼

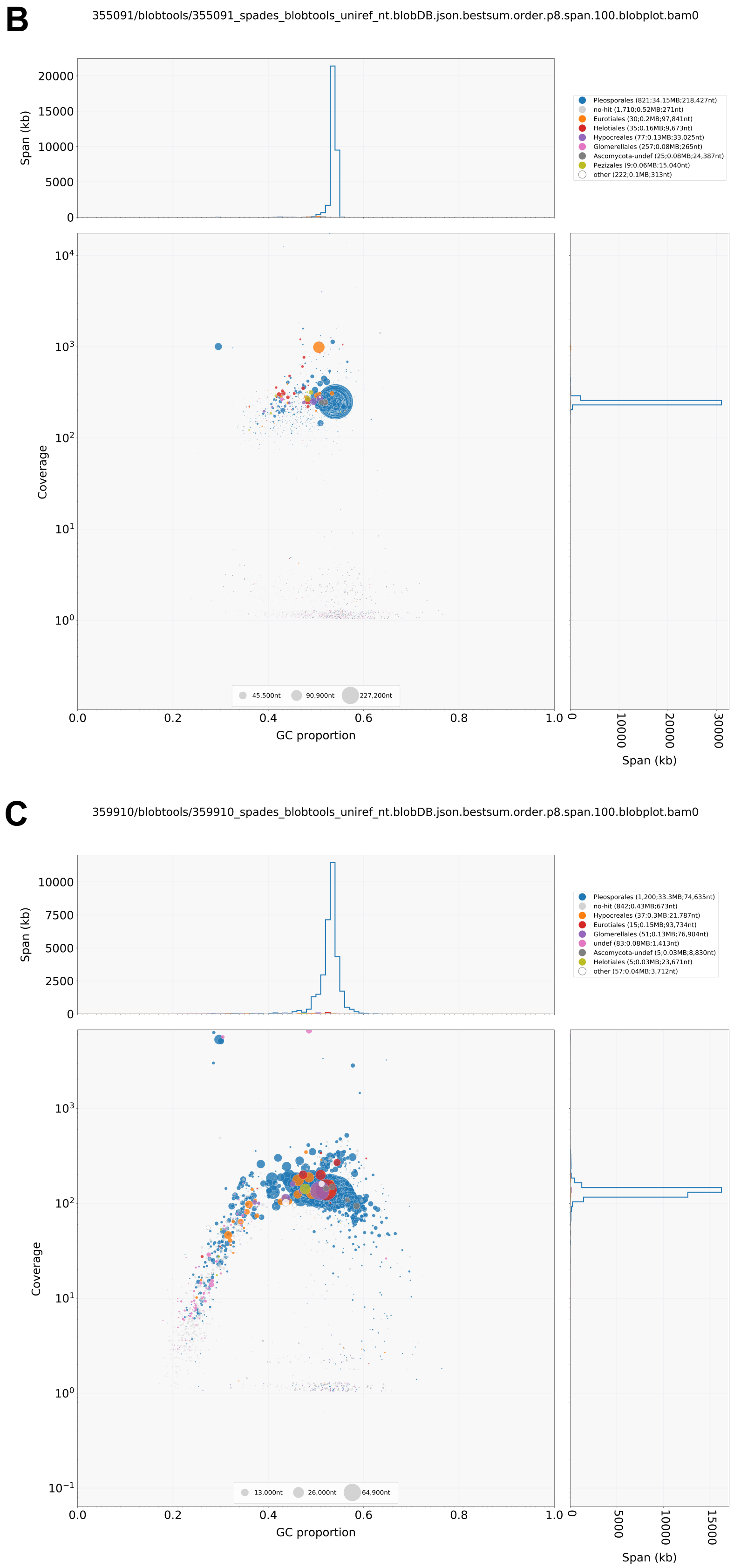

Supplementary Figure 2: continued. ▼

D

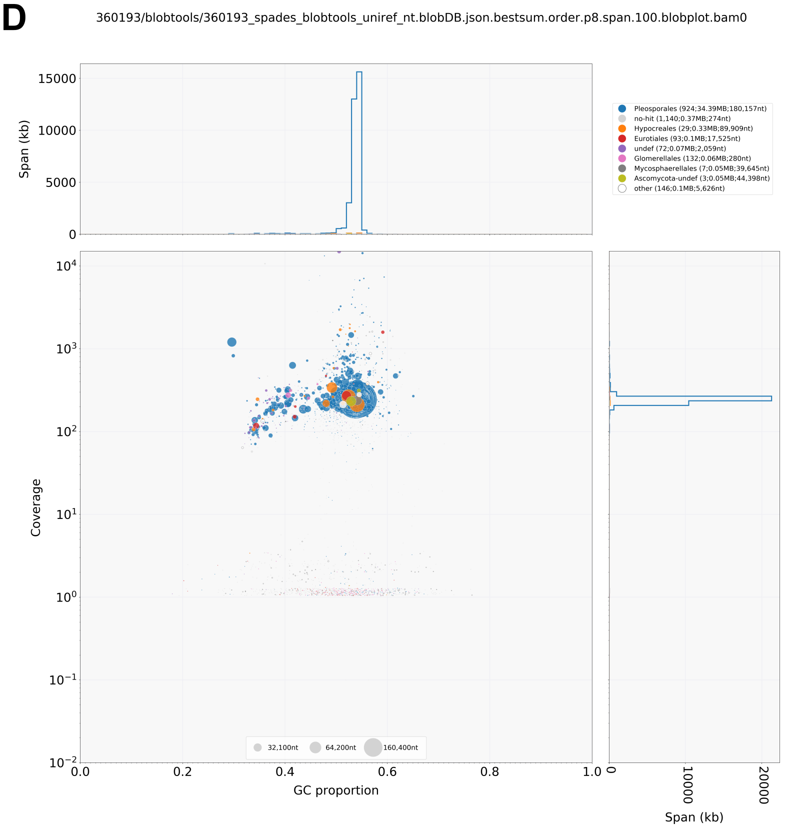

E

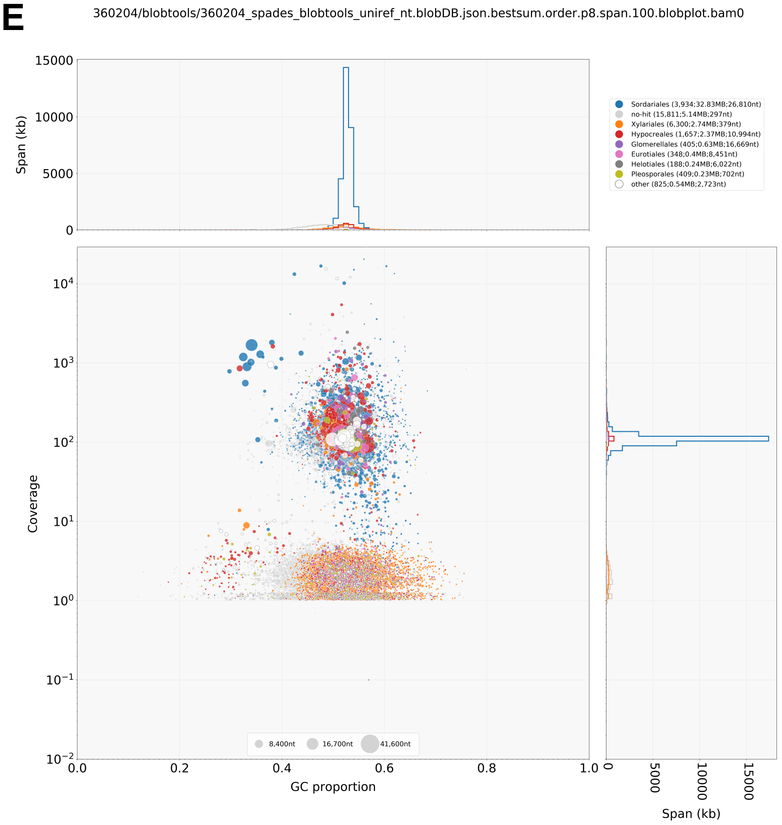

Supplementary Figure 2: continued. ▼

F

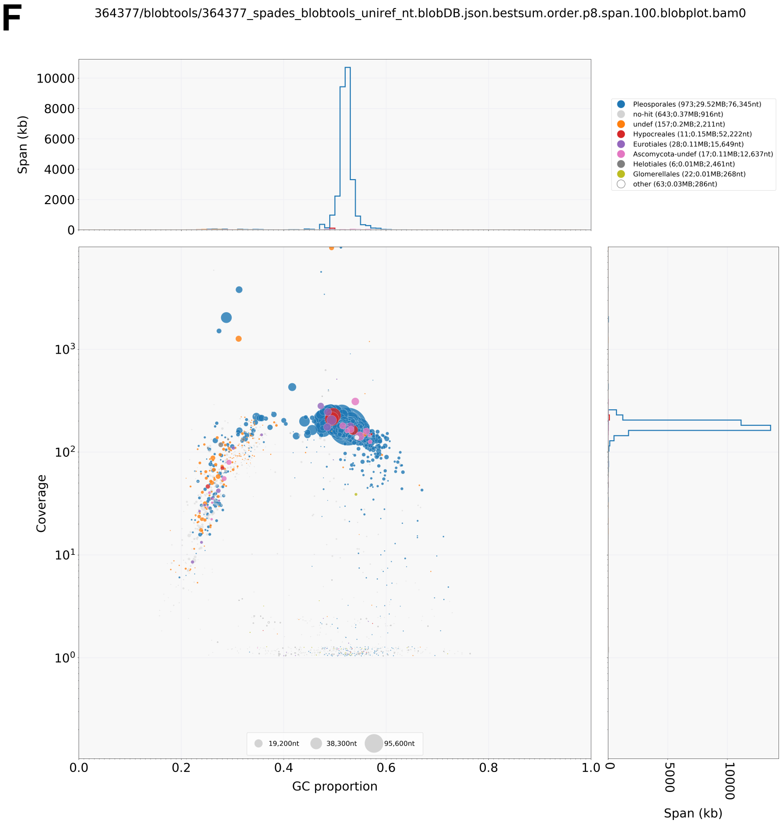

G

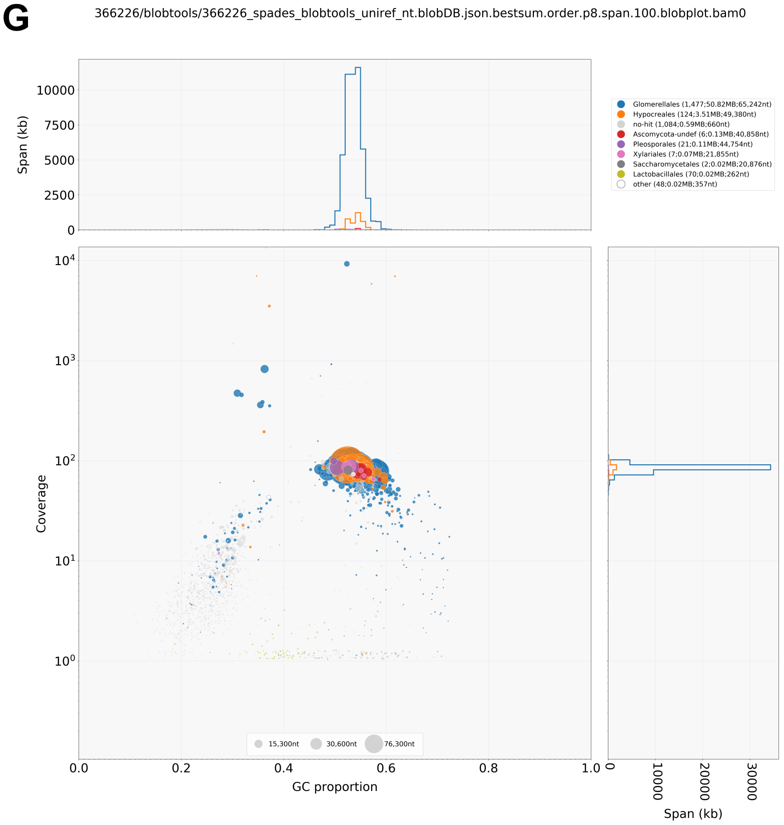

Supplementary Figure 2: continued. ▼

H

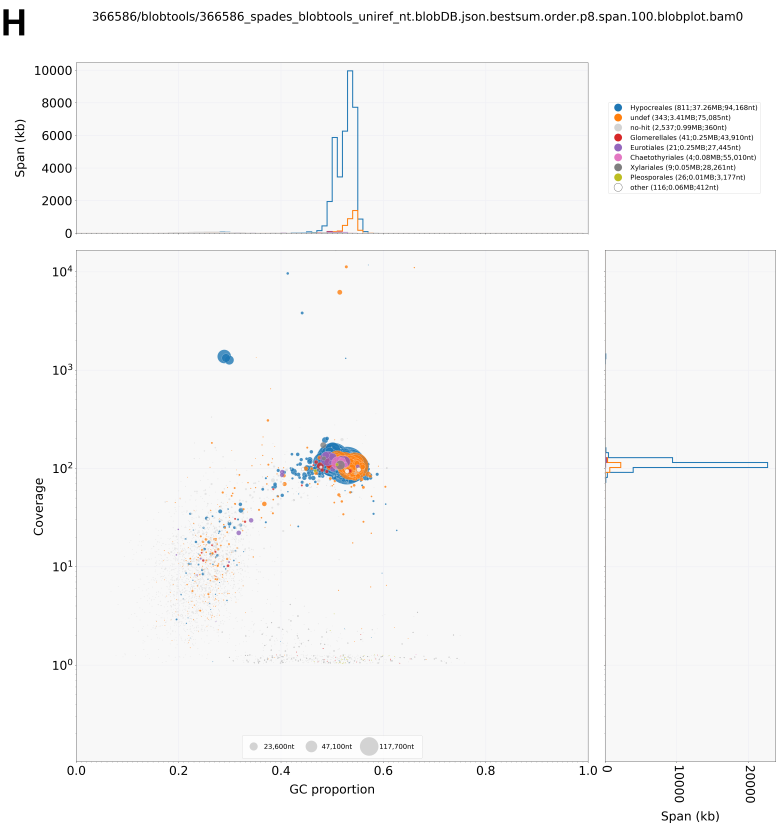

I

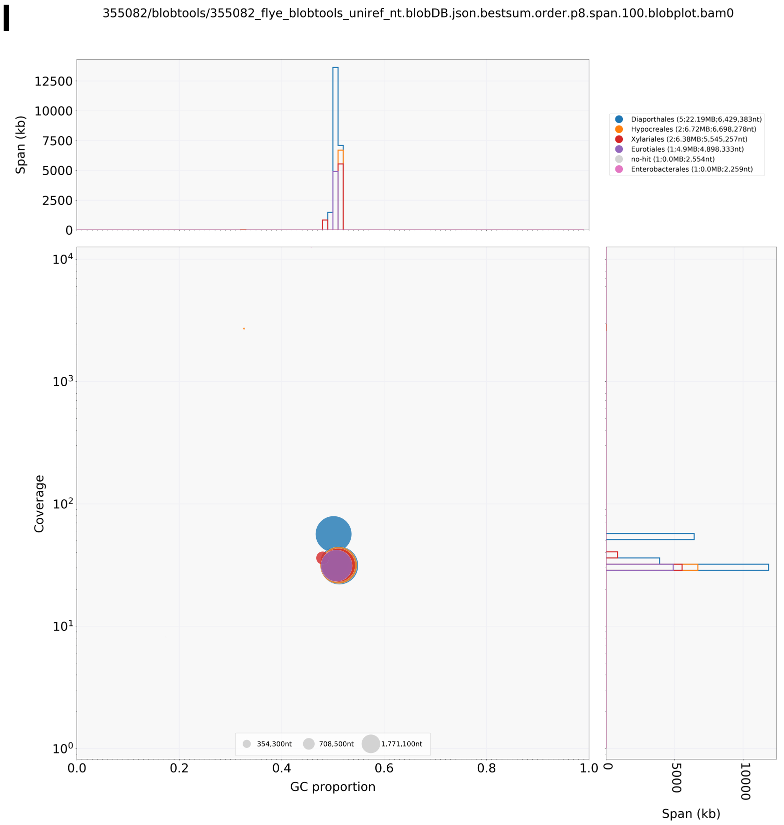

Supplementary Figure 2: continued. ▼

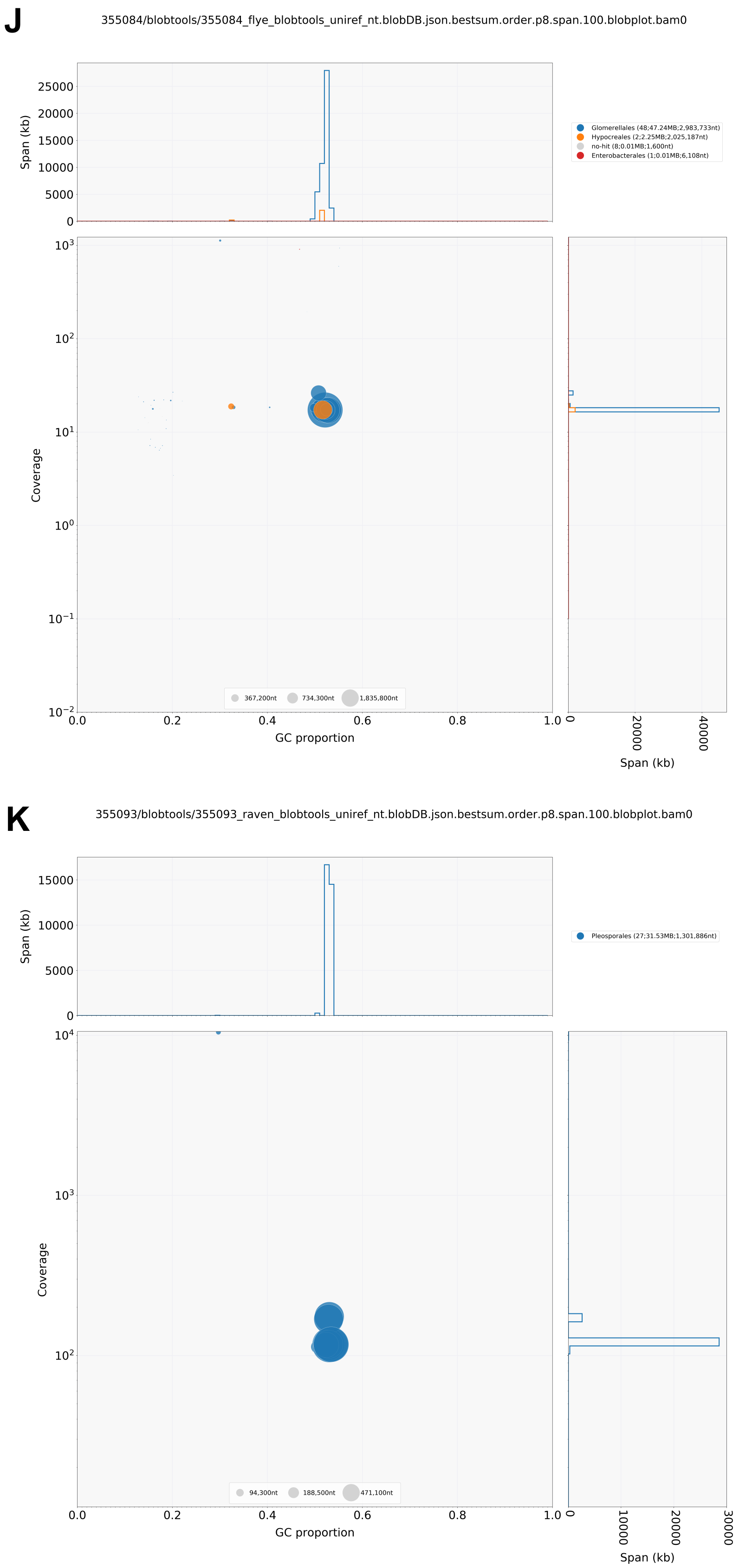

Supplementary Figure 2: continued. ▼

L

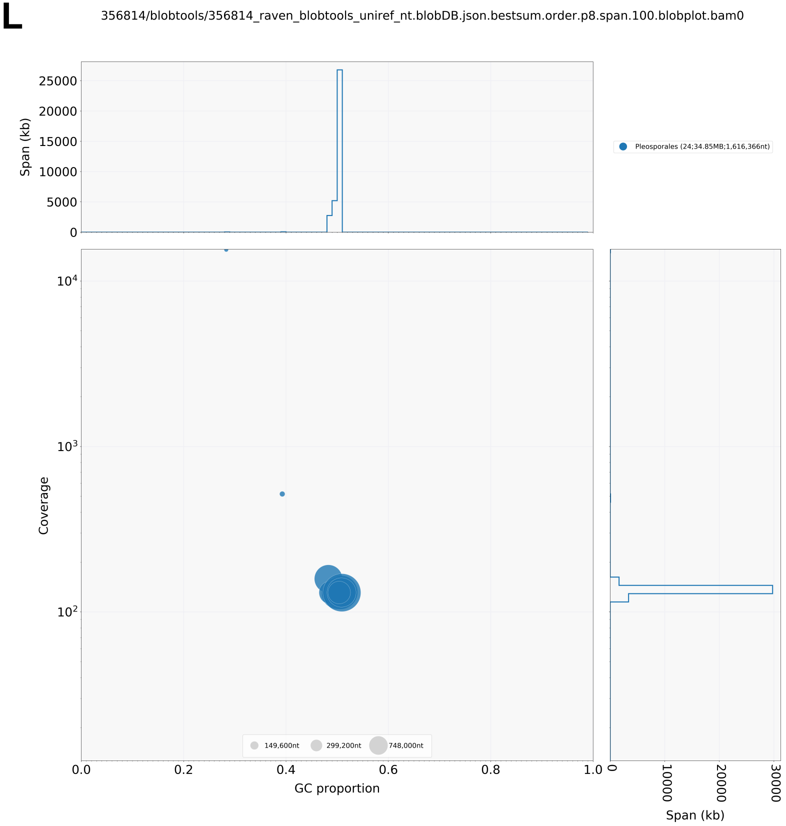

M

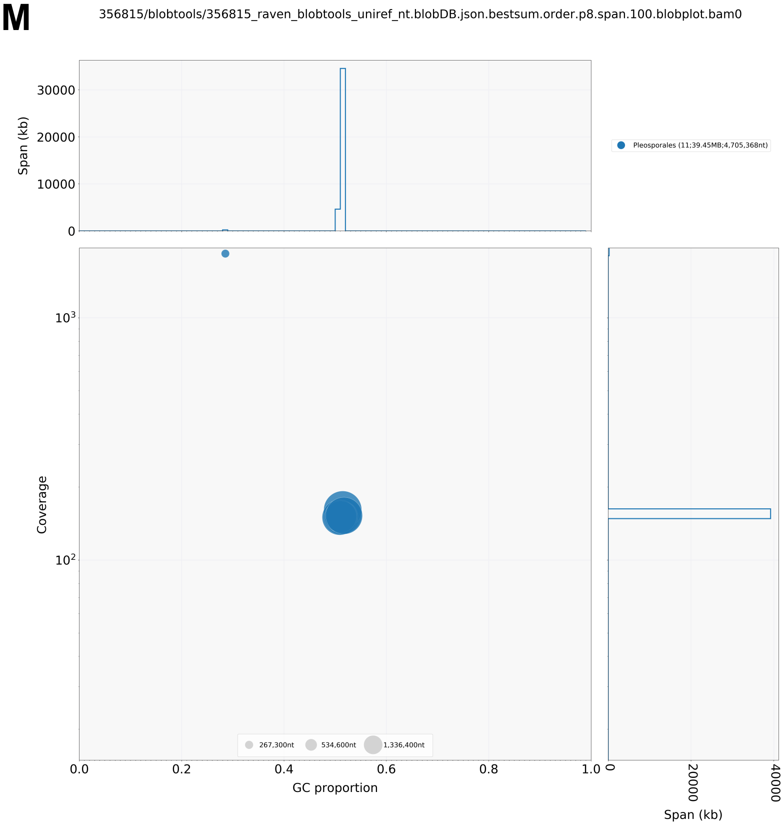

Supplementary Figure 2: continued. ▼

N

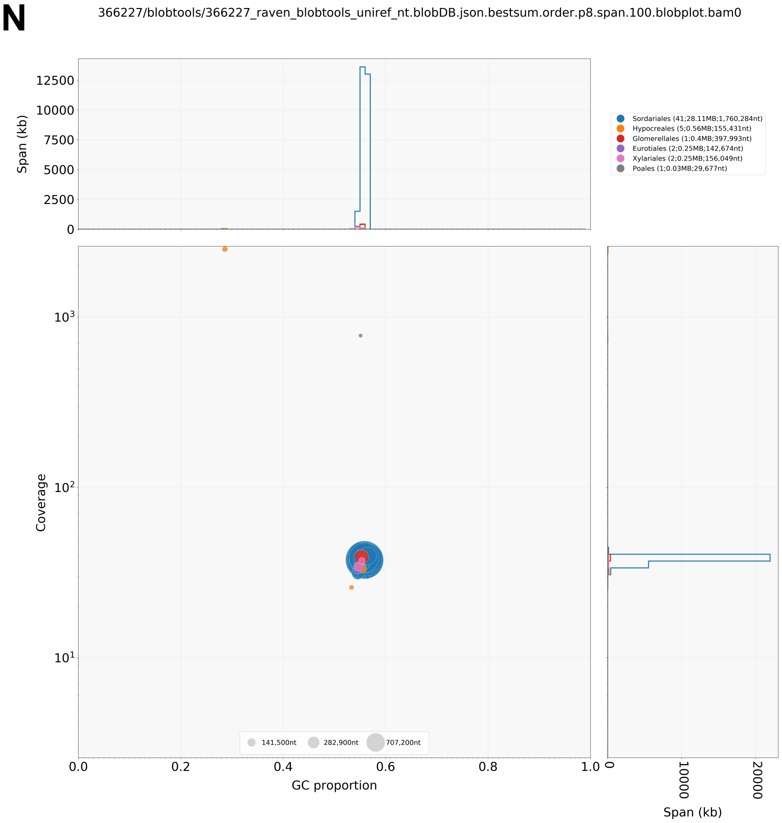

O

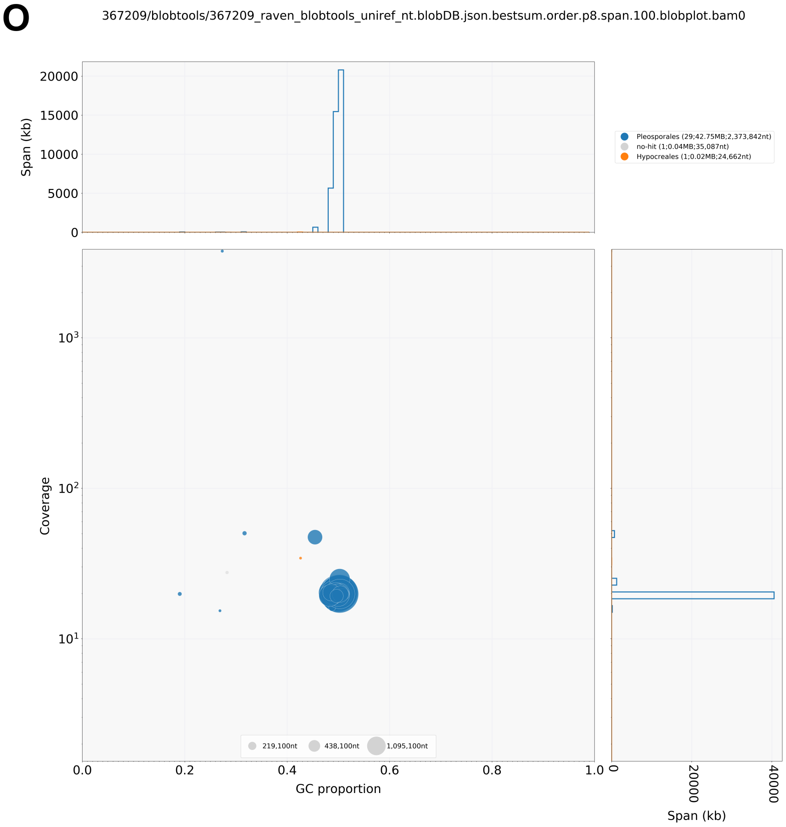

Supplementary Figure 2: continued.

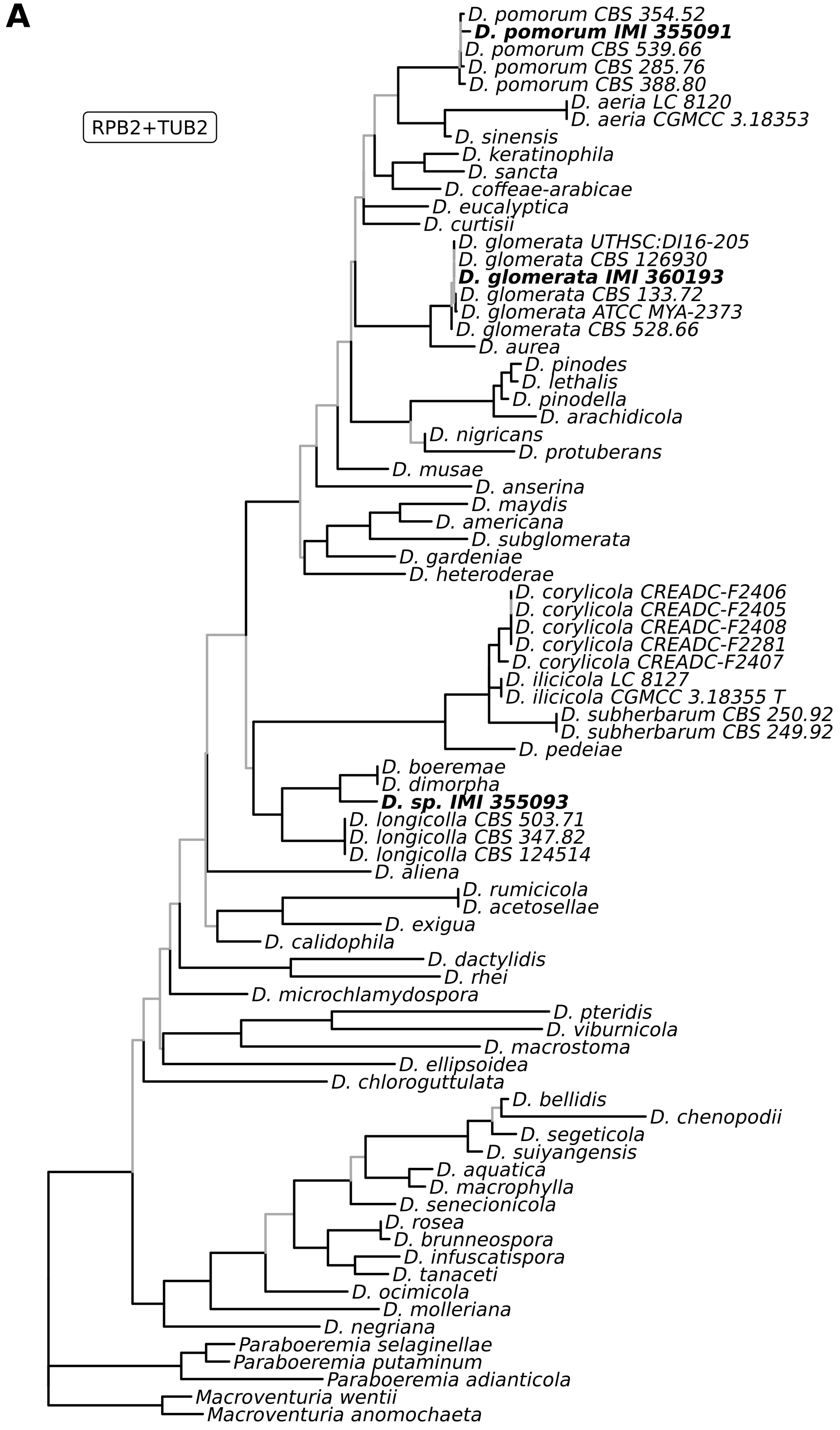

Supplementary Figure 3: ML phylogenies produced using RAxML to refine classification of the 15 endophyte strains (shown in bold). Branches with significant bootstrap support (≥ 70) are in black, while others are in grey. The genetic markers used to build each tree are shown in the top left. **(A)** *D.* = *Didymella.* ▼

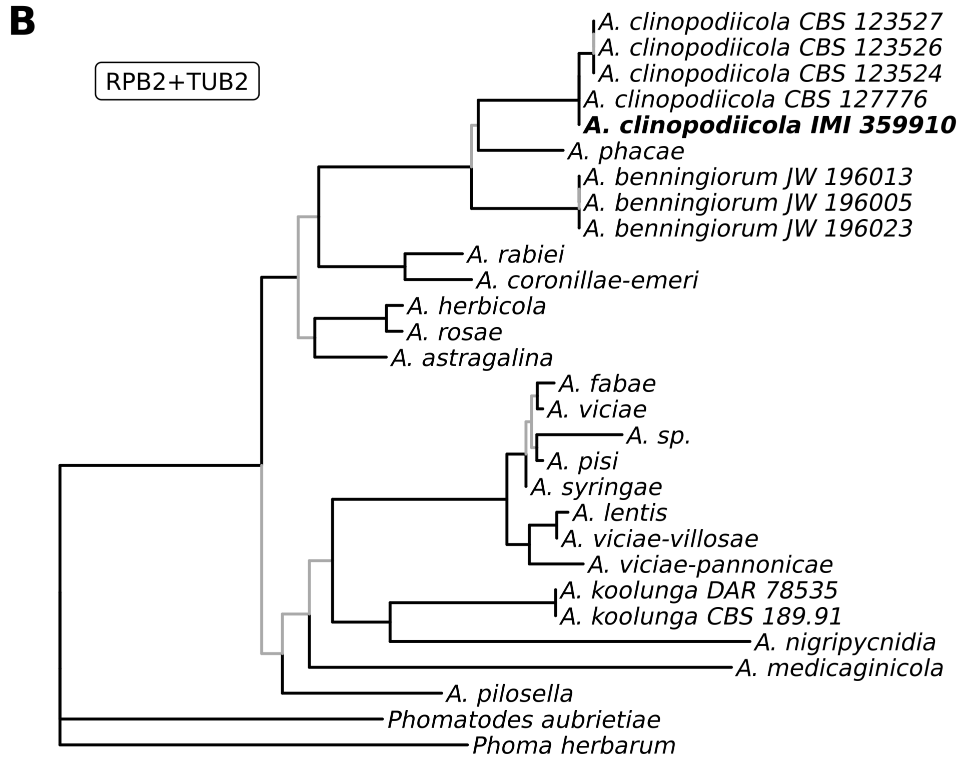

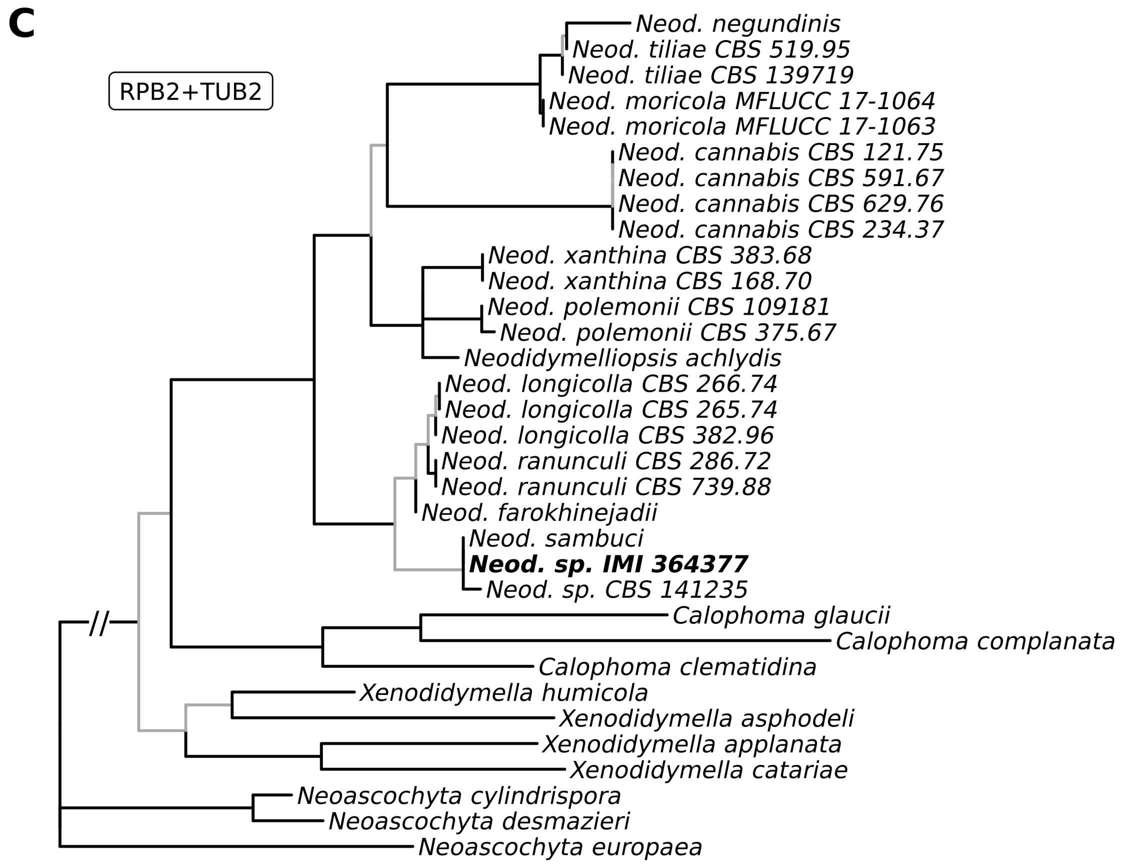

Supplementary Figure 3: continued. **(B)** *A.* = *Ascochyta* **(C)** *Neod.* = *Neodidymelliopsis.* ▼

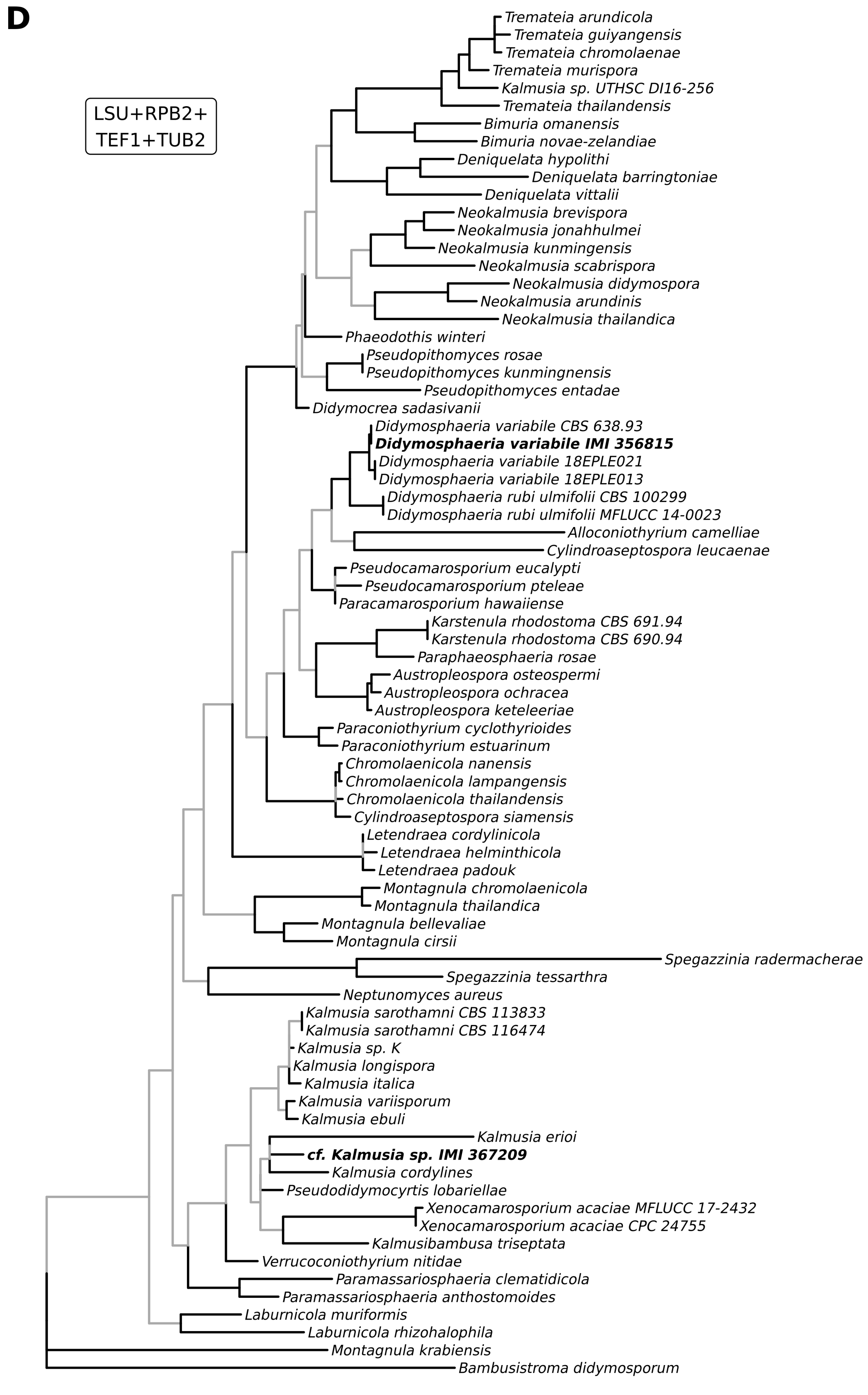

Supplementary Figure 3: continued. **(D)** *Didymosphaeriaceae.* ▼

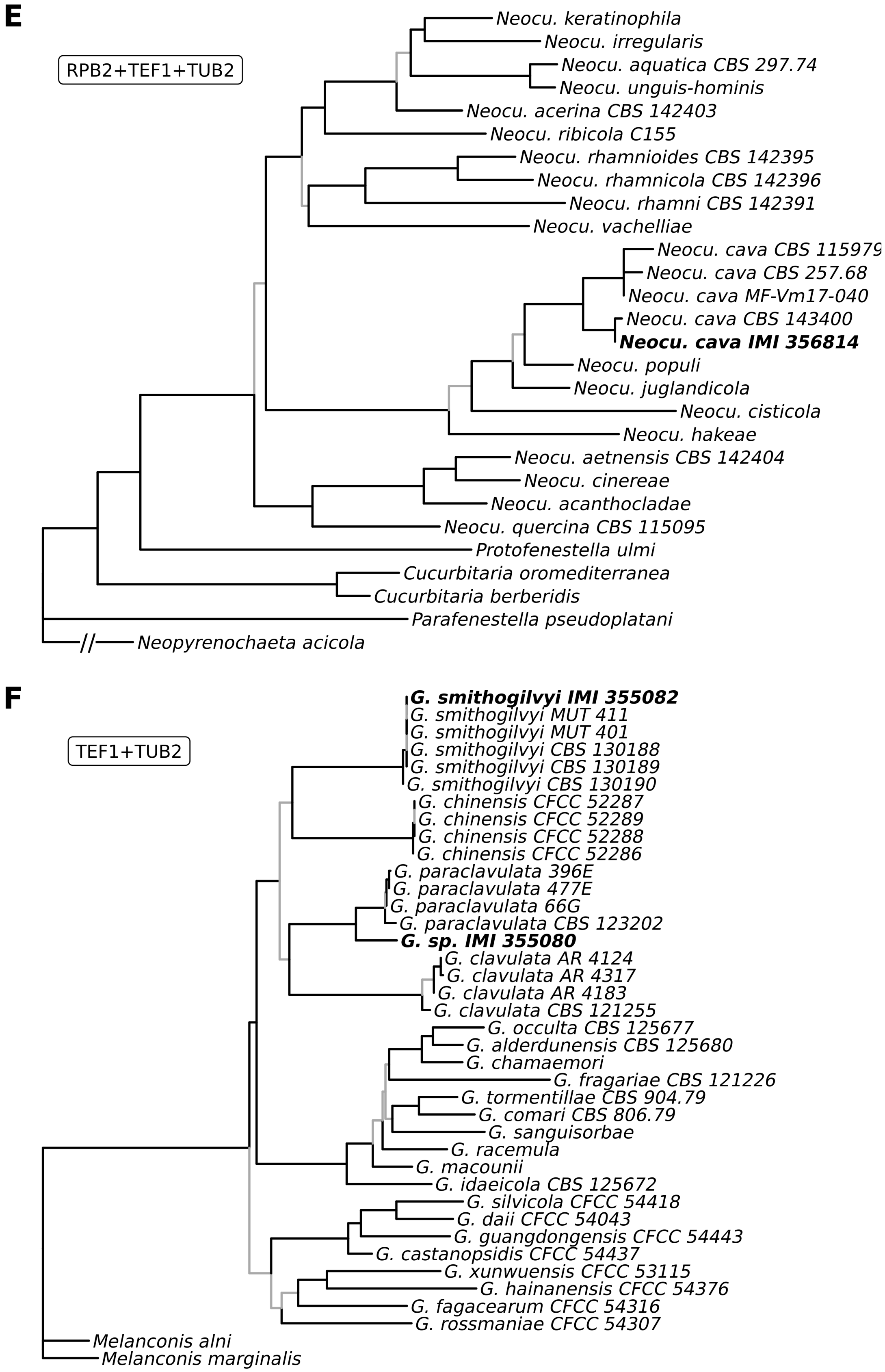

Supplementary Figure 3: continued. **(E)** *Neocu.* = *Neocucurbitaria* **(F)** *G.* = *Gnomoniopsis*. ▼

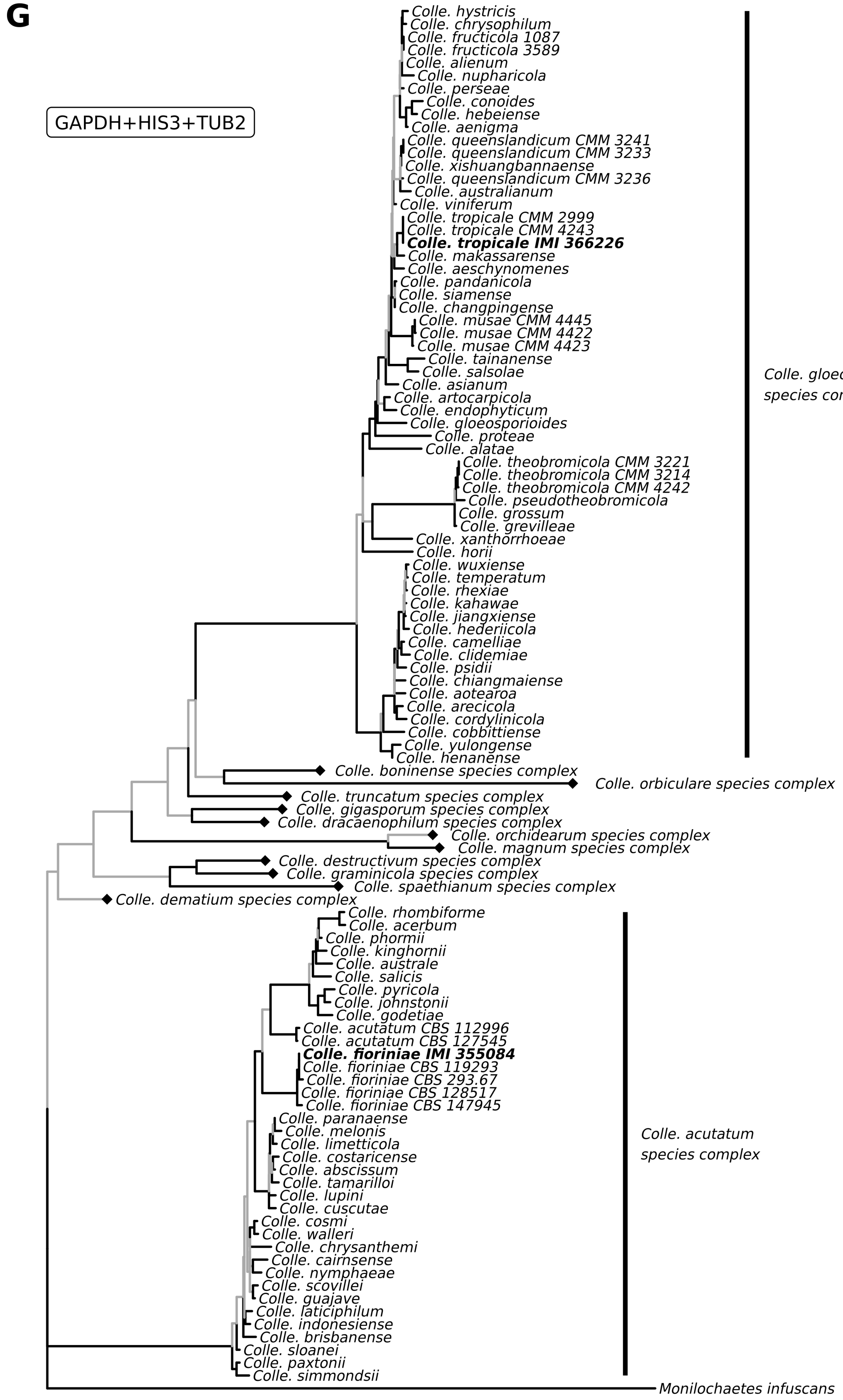

Supplementary Figure 3: continued. **(G)** *Colle.* = *Colletotrichum*. Diamonds indicate collapsed species complexes. ▼

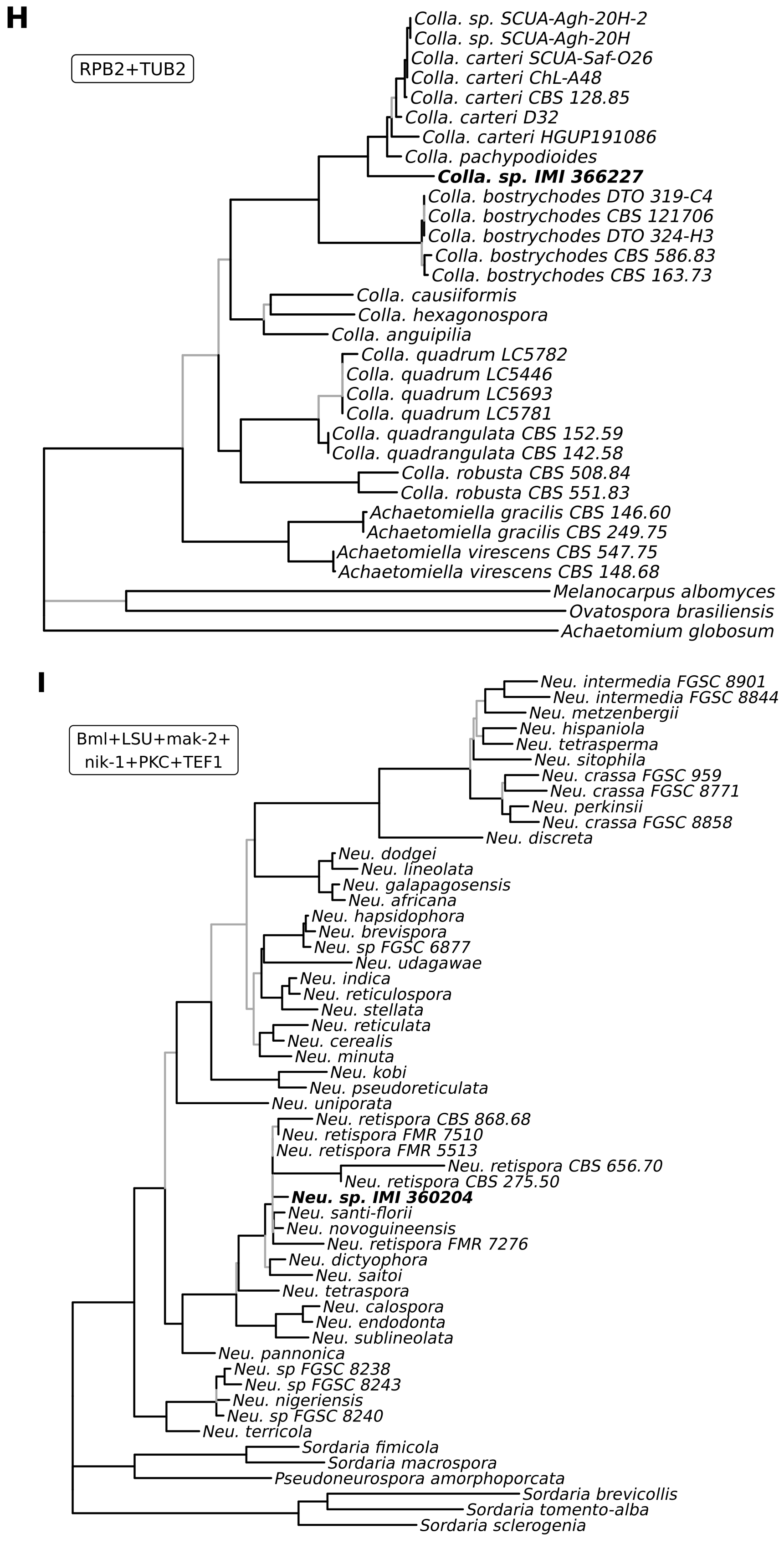

Supplementary Figure 3: continued. **(H)** *Colla.* = *Collariella* **(I)** *Neu.* = *Neurospora.* ▼

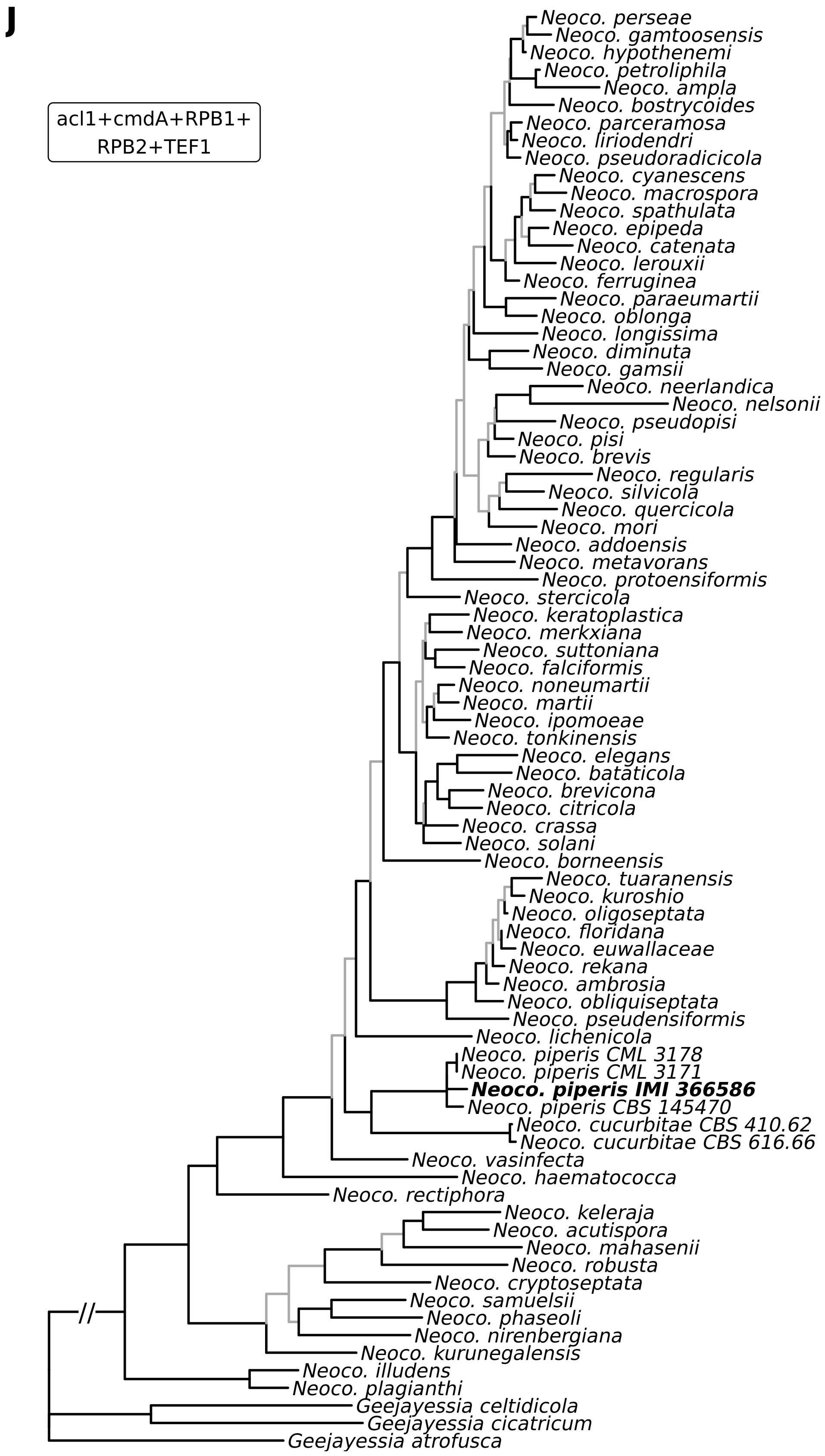

Supplementary Figure 3: continued. **(J)** *Neoco.* = *Neocosmospora*.

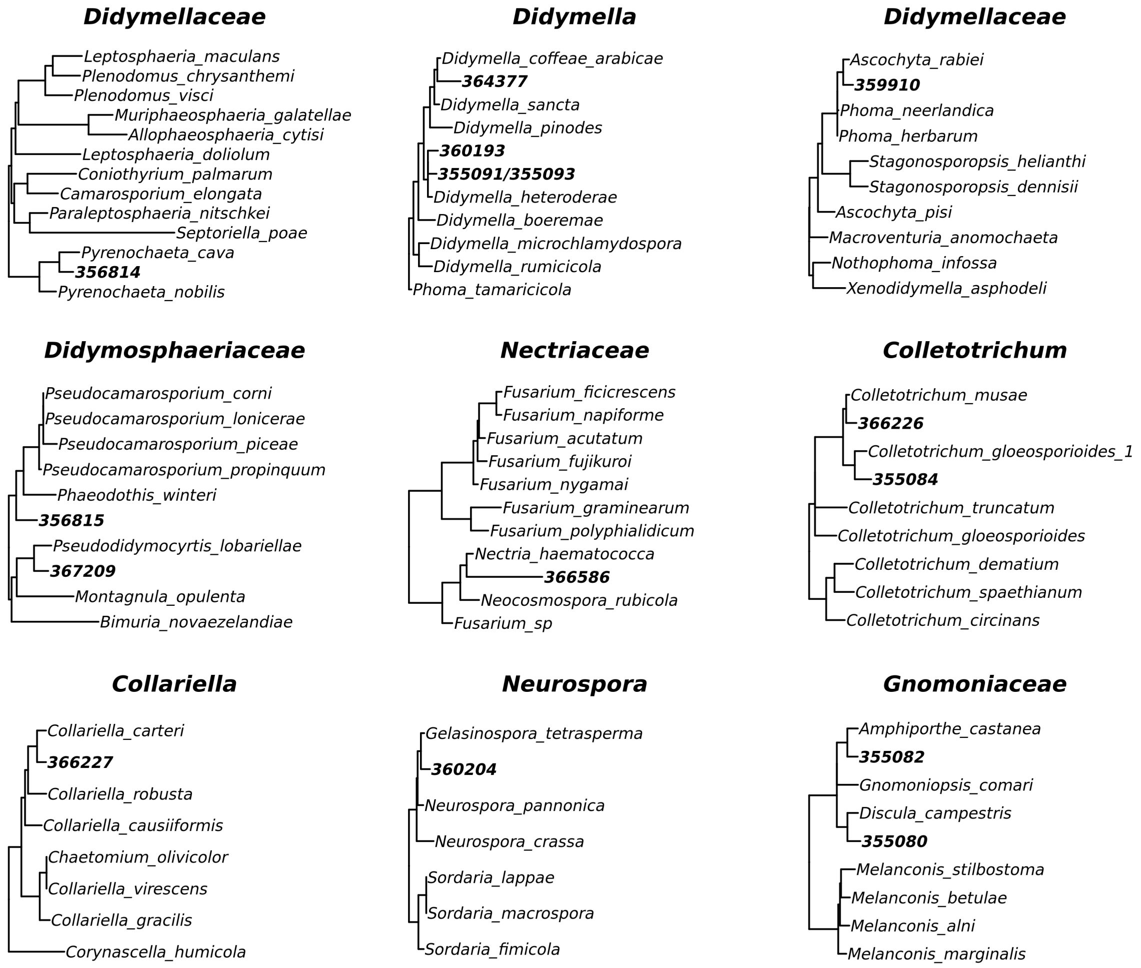

Supplementary Figure 4: T-BAS placements for the 15 endophyte strains. For visual clarity, clades containing our strains were extracted from the T-BAS tree and are shown separately. Due to high relatedness, IMI 355091 and IMI 355093 were grouped into a single branch in *Didymella* by T-BAS. *Pyrenochaeta cava* = *Neocucurbitaria cava*; *Gelasinospora tetrasperma* = *Neurospora tetraspora*.

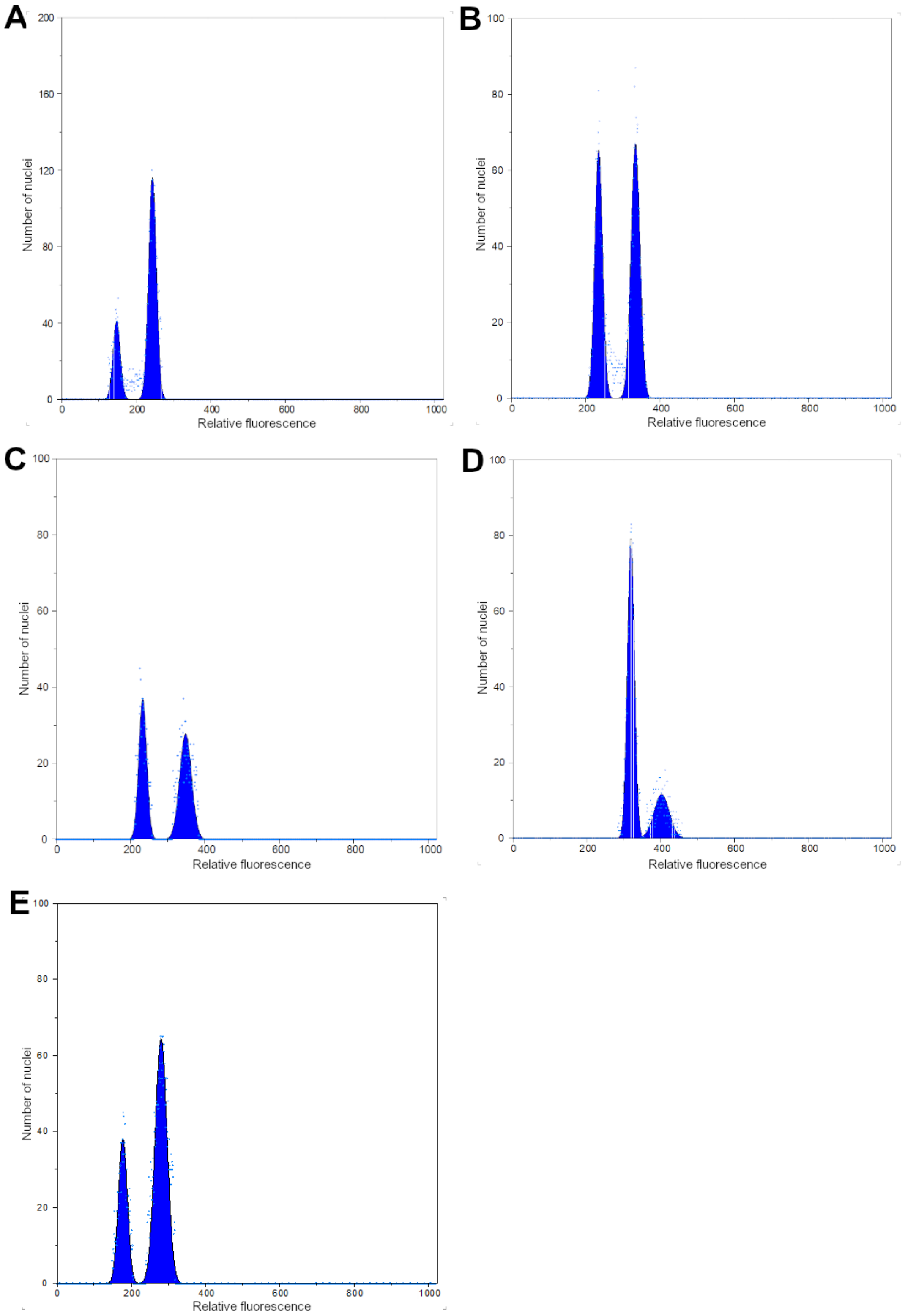

Supplementary Figure 5: Flow cytometry histograms showing the relative fluorescence of fungal nuclei from the sample and calibration standard. One representative histogram is shown out of the total three runs made per sample. In all cases the left-hand peak is the sample while the right-hand peak is the standard. **(A)** IMI 355093 **(B)** IMI 356814 **(C)** IMI 359910 **(D)** IMI 360204 **(E)** IMI 364377.

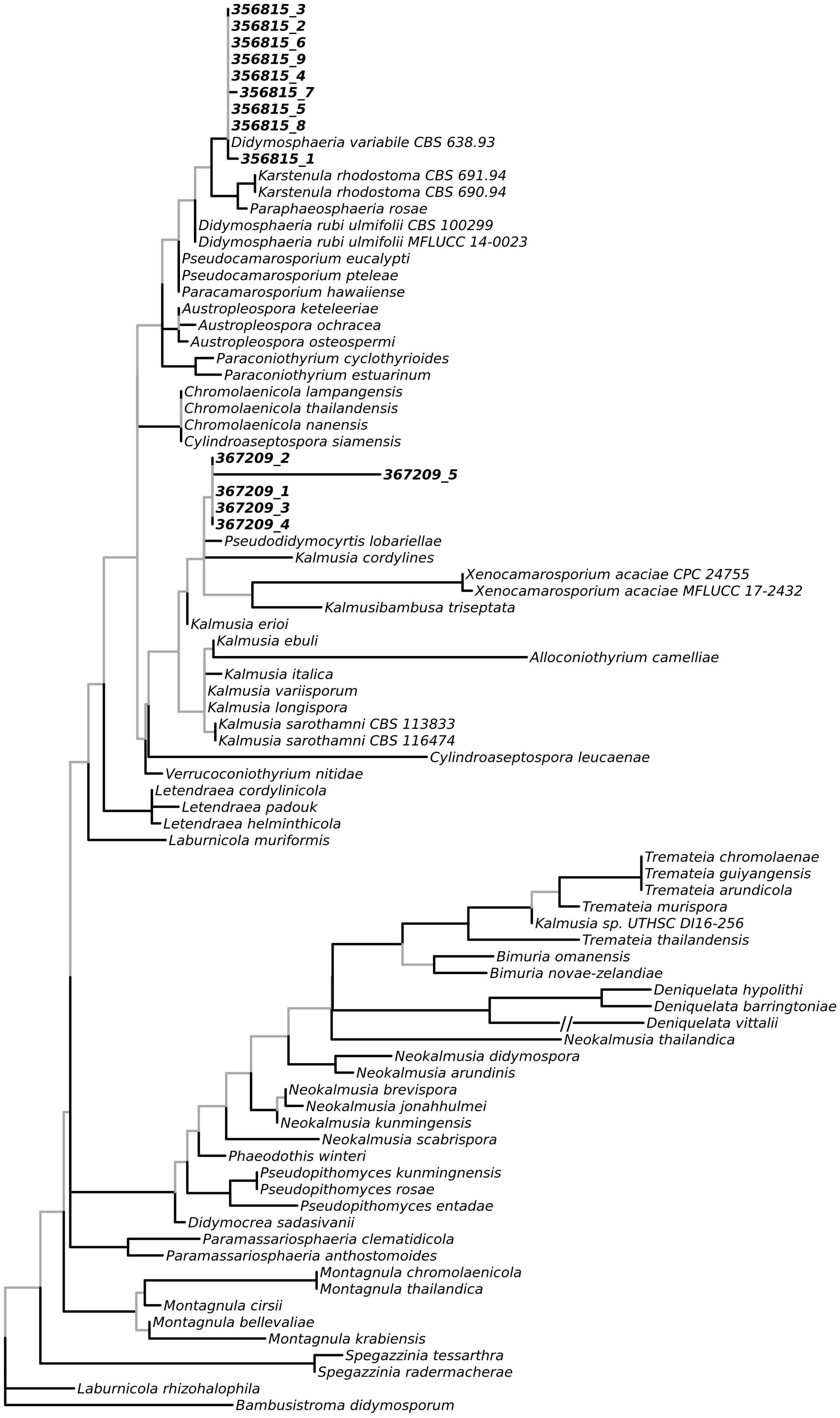

Supplementary Figure 6: LSU gene tree of the *Didymosphaeriaceae* produced using RAxML. Branches with significant bootstrap support (≥ 70) are in black, while others are in grey. Multiple copies of LSU from strains IMI 356815 and IMI 367209 are shown in bold.

References

Baker, S. E. et al. (2015). Draft genome sequence of *Neurospora crassa* strain FGSC 73. *Genome Announcements* 3(2):e00074–15. DOI: 10.1128/genomeA.00074-15.

Baroncelli, R. et al. (2022). Genome evolution and transcriptome plasticity associated with adaptation to monocot and eudicot plants in *Colletotrichum* fungi. *bioRxiv [Preprint]*. DOI: 10.1101/2022.09.22.508453.

Bourne, E. C. et al. (2014). Large and variable genome size unrelated to serpentine adaptation but supportive of cryptic sexuality in *Cenococcum geophilum*. *Mycorrhiza* 24:13–20. DOI: 10.1007/s00572-013-0501-3.

Chen, Q. et al. (2017). *Didymellaceae* revisited. *Studies in Mycology* 87:105–159. DOI: 10.1016/j.simyco.2017.06.002.

Crous, P. W., Lombard, L., et al. (2021). *Fusarium*: more than a node or a foot-shaped basal cell. *Studies in Mycology* 98:100116. DOI: 10.1016/j.simyco.2021.100116.

Crous, P. W., Schumacher, R. K., et al. (2019). New and Interesting Fungi. 2. *Fungal Systematics and Evolution* 3:57–134. DOI: 10.3114/fuse.2019.03.06.

Doležel, J., Binarová, P., and Lucretti, S. (1989). Analysis of Nuclear DNA Content in Plant Cells by Flow Cytometry. *Biologia Plantarum* 31(2):113–120. DOI: 10.1007/BF02907241.

Doležel, J., Bartoš, J., et al. (2003). Nuclear DNA content and genome size of trout and human. *Cytometry Part A* 51A(2):127–128. DOI: 10.1002/cyto.a.10013.

Grigoriev, I. V. et al. (2014). MycoCosm portal: gearing up for 1000 fungal genomes. *Nucleic Acids Research* 42:699–704. DOI: 10.1093/nar/gkt1183.

Haridas, S. et al. (2020). 101 *Dothideomycetes* genomes: A test case for predicting lifestyles and emergence of pathogens. *Studies in Mycology* 96:141–153. DOI: 10.1016/j.simyco.2020.01.003.

Hou, L. W. et al. (2020). The phoma-like dilemma. *Studies in Mycology* 96:309–396. DOI: 10.1016/j.simyco.2020.05.001.

Hyde, K. D. et al. (2019). Fungal diversity notes 1036-1150: taxonomic and phylogenetic contributions on genera and species of fungal taxa. *Fungal Diversity* 96:1–242. DOI: 10.1007/s13225-019-00429-2.

Jaklitsch, W. M. et al. (2018). A preliminary account of the *Cucurbitariaceae*. *Studies in Mycology* 90:71–118. DOI: 10.1016/j.simyco.2017.11.002.

Jiang, N. et al. (2021). Morphology and phylogeny of *Gnomoniopsis* (*Gnomoniaceae, Diaporthales*) from *Fagaceae* leaves in China. *Journal of Fungi* 7:792. DOI: 10.3390/jof7100792.

Karácsony, Z. et al. (2021). The fungus *Kalmusia longispora* is able to cause vascular necrosis on *Vitis vinifera*. *PLoS ONE* 16(10):e0258043. DOI: 10.1371/journal.pone.0258043.

Liu, F. et al. (2022). Updating species diversity of *Colletotrichum*, with a phylogenomic overview. *Studies in Mycology* 101:1–56. DOI: 10.3114/sim.2022.101.01.

Mesny, F. et al. (2021). Genetic determinants of endophytism in the *Arabidopsis* root mycobiome. *Nature Communications* 12:7227. DOI: 10.1038/s41467-021-27479-y.

Nygren, K. et al. (2011). A comprehensive phylogeny of *Neurospora* reveals a link between reproductive mode and molecular evolution in fungi. *Molecular Phylogenetics and Evolution* 59(3):649–663. DOI: 10.1016/j.ympev.2011.03.023.

Scarpari, M. et al. (2020). *Didymella corylicola* sp. nov., a new fungus associated with hazelnut fruit development in Italy. *Mycological Progress* 19:317–328. DOI: 10.1007/s11557-020-01562-y.

Valenzuela-Lopez, N. et al. (2018). Coelomycetous *Dothideomycetes* with emphasis on the families *Cucurbitariaceae* and *Didymellaceae*. *Studies in Mycology* 90:1–69. DOI: 10.1016/j.simyco.2017.11.003.

Vieira, W. A. d. S. et al. (2020). Optimal markers for the identification of *Colletotrichum* species. *Molecular Phylogenetics and Evolution* 143:106694. DOI: 10.1016/j.ympev.2019.106694.

Wanasinghe, D. N., Phookamsak, R., et al. (2017). A family level rDNA based phylogeny of Cucurbitariaceae and Fenestellaceae with descriptions of new *Fenestella* species and *Neocucurbitaria* gen. nov. *Mycosphere* 8(4):397–414. DOI: 10.5943/mycosphere/8/4/2.

Wanasinghe, D. N. and Mortimer, P. E. (2022). Taxonomic and Phylogenetic Insights into Novel *Ascomycota* from Forest Woody Litter. *Biology* 11:889. DOI: 10.3390/biology11060889.

Wang, X. W., Han, P. J., et al. (2022). Taxonomy, phylogeny and identification of *Chaetomiaceae* with emphasis on thermophilic species. *Studies in Mycology* 101:121–243. DOI: 10.3114/sim.2022.101.03.

Wang, X. W., Houbraken, J., et al. (2016). Diversity and taxonomy of *Chaetomium* and chaetomium-like fungi from indoor environments. *Studies in Mycology* 84:145–224. DOI: 10.1016/j.simyco.2016.11.005.
